# Shallow Univariate ReLU Networks as Splines: Initialization, Loss Surface, Hessian, and Gradient Flow Dynamics

**DOI:** 10.3389/frai.2022.889981

**Published:** 2022-05-11

**Authors:** Justin Sahs, Ryan Pyle, Aneel Damaraju, Josue Ortega Caro, Onur Tavaslioglu, Andy Lu, Fabio Anselmi, Ankit B. Patel

**Affiliations:** ^1^Department of Neuroscience, Baylor College of Medicine, Houston, TX, United States; ^2^Department of Electrical Engineering, Rice University, Houston, TX, United States; ^3^Department of Computational and Applied Mathematics, Rice University, Houston, TX, United States

**Keywords:** neural networks, symmetry, implicit bias, splines, learning dynamics

## Abstract

Understanding the learning dynamics and inductive bias of neural networks (NNs) is hindered by the opacity of the relationship between NN parameters and the function represented. Partially, this is due to symmetries inherent within the NN parameterization, allowing multiple different parameter settings to result in an identical output function, resulting in both an unclear relationship and redundant degrees of freedom. The NN parameterization is invariant under two symmetries: permutation of the neurons and a continuous family of transformations of the scale of weight and bias parameters. We propose taking a quotient with respect to the second symmetry group and reparametrizing ReLU NNs as continuous piecewise linear splines. Using this spline lens, we study learning dynamics in shallow univariate ReLU NNs, finding unexpected insights and explanations for several perplexing phenomena. We develop a surprisingly simple and transparent view of the structure of the loss surface, including its critical and fixed points, Hessian, and Hessian spectrum. We also show that standard weight initializations yield very flat initial functions, and that this flatness, together with overparametrization and the initial weight scale, is responsible for the strength and type of implicit regularization, consistent with previous work. Our implicit regularization results are complementary to recent work, showing that initialization scale critically controls implicit regularization via a kernel-based argument. Overall, removing the weight scale symmetry enables us to prove these results more simply and enables us to prove new results and gain new insights while offering a far more transparent and intuitive picture. Looking forward, our quotiented spline-based approach will extend naturally to the multivariate and deep settings, and alongside the kernel-based view, we believe it will play a foundational role in efforts to understand neural networks. Videos of learning dynamics using a spline-based visualization are available at http://shorturl.at/tFWZ2.

## 1. Introduction

Deep learning has revolutionized the field of machine learning (ML), leading to state of the art performance in image segmentation, medical imaging, machine translation, chess playing, reinforcement learning, and more. Despite being intensely studied and widely used, theoretical understanding of some of its fundamental properties remains poor. One critical, yet mysterious property of deep learning is the root cause of the excellent generalization achieved by overparameterized networks (Zhang et al., 2016).

In contrast to other machine learning techniques, continuing to add parameters to a deep network (beyond zero training loss) tends to *improve* generalization performance. This has even been observed for networks that are massively overparameterized, wherein, according to traditional ML theory, they should (over)fit the training data (Neyshabur et al., 2015). This overparameterization leads to a highly complicated loss surface, with increasingly many local minima. How does training networks with excess capacity lead to generalization? And how can generalization error decrease with overparameterization? How can gradient descent in a loss landscape riddled with local minima so frequently converge to near-global minima? One possible solution is a phenomenon known as implicit regularization (or implicit bias). Empirical studies (Zhang et al., 2016; Advani et al., 2020; Geiger et al., 2020) have shown that overparametrized NNs behave *as if* they possess strong regularization, even when trained from scratch with no explicit regularization—instead possessing *implicit* regularization or bias (IR or IB), since such regularization is *not* explicitly imposed by the designer.

We approach these issues by considering symmetries of the NN parameterization. Several recent works have brought attention to the importance of symmetries to deep learning (Badrinarayanan et al., 2015; Kunin et al., 2020; Tayal et al., 2020). One important advance is building symmetry aware architectures that automatically generalize, such as convolutional layers granting translation-invariant representations, or many other task-specific symmetries (Liu et al., 2016; Barbosa et al., 2021; Bertoni et al., 2021; Liu and Okatani, 2021). Understanding and exploiting symmetries may lead to similar improvements, resulting in better performance or models that require less training data due to implementation of expert-knowledge based symmetry groups.

We consider a new spline-based reparamaterization of a shallow ReLU NN, explicitly based on taking a quotient with respect to an existing weight symmetry. In particular, we focus on shallow fully connected univariate ReLU networks, whose parameters will always result in a Continuous Piecewise Linear (CPWL) output. We reparameterize the CPWL function implemented by the network in terms of the locations of the nonlinearities (*breakpoints*), the associated change in slope (*delta-slopes*), and a parameter that identifies which side of the breakpoint the associated neuron is active on (*orientation*). This parameterization is defined formally in section 2.1, and illustrated in **Figure 2**. We provide theoretical results for shallow networks, with experiments confirming these results.

### 1.1. Main Contributions

The main contribution of this work are as follows:

- *Initialization: Increasingly Flat with Width*. In the spline perspective, once the weight symmetry has been accounted for, neural network parameters determine the locations of breakpoints and their delta-slopes in the CPWL reparametrization. We prove that, for common initializations, these distributions are mean 0 with low standard deviation. Notably, the delta-slope distribution becomes increasingly concentrated near 0 as the width of the network increases, leading to flatter initial approximations.
- *A characterization of the Loss Surface, Critical Points and Hessian, revealing that Flat Minima are due to degeneracy of breakpoints*. We fully characterize the Hessian of the loss surface at critical points, revealing that the Hessian is positive semi-definite, with 0 eigenvalues occurring when elements of its Gram matrix set are linearly dependent. We show that many of these 0 eigenvalues occur due to symmetry artifacts of the standard NN parametrization. We characterize the ways this can occur, including whenever multiple breakpoints share the same active data—thus we expect that at any given critical point in an overparametrized network, the loss surface will be flat in many directions.
- *Implicit Regularization is due to Flat Initialization in the Overparametrized Regime*. We find that implicit regularization in overparametrized Fully Connected (FC) ReLU nets is due to three factors: (i) the very flat initialization, (ii) the curvature-based parametrization of the approximating function (breakpoints and delta-slopes, made explicitly clear by our symmetry-quotiented reparametrization) and (iii) the role of gradient descent (GD) in preserving initialization and providing regularization of curvature. In particular, each neuron has an global, rather than local, impact as each contributes an affine ReLU. Thus, backpropogation distributes the “work” of fitting the training data over many units, leading to regularized delta-slope and breakpoints. All else equal, these non-local effects mean that more overparametrization leads to each unit mattering less, thus typically resulting in better generalization due to implicit regularization (Neyshabur et al., 2015, 2018).

## 2. Theoretical Results

### 2.1. Spline Parametrization and Notation

Consider a fully connected ReLU neural net $\hat{f}\left(x;\theta \right)$ with a single hidden layer of width *H*, scalar input *x* ∈ ℝ and scalar output ŷ ∈ ℝ, which we are attempting to match to a target function *f*(*x*), from which we have *N* sample input/output data pairs (*x*_*n*_, *y*_*n*_); the full vectors of inputs and outputs are denoted x and y, respectively. $\hat{f}\left(\cdot ;\theta \right)$ is a continuous piecewise linear (CPWL) function since the ReLU nonlinearity is CPWL.


$$\hat{f}\left(x;\theta_{\text{NN}}\right)≜b_{0}+\overset{H}{\underset{i=1}{\sum }}v_{i}{\left(w_{i}x+b_{i}\right)}_{+}$$

Here the NN parameters $\theta_{\text{NN}}≜\left\{b_{0}\right\}\cup {\left\{\left(w_{i},b_{i},v_{i}\right)\right\}}_{i=1}^{H}$ denote the global bias plus the input weight, bias, and output weight, respectively of neuron *i*, and (·)_+_ ≜ max {0, ·} denotes the ReLU function.

**Figure 1:**
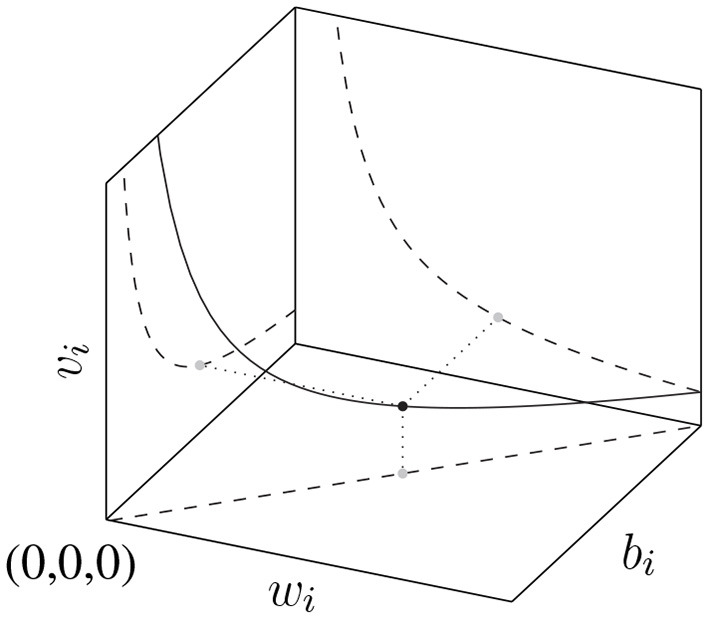
The manifold generated by the scaling transformation of input weight, input bias, output weight: $\left(w_{i},b_{i},v_{i}\right)\mapsto \left(\alpha_{i}w_{i},\alpha_{i}b_{i},\alpha_{i}^{-1}v_{i}\right)$, which leaves $\hat{f}\left(x\right)$ invariant.

An important observation is that the ReLU NN parametrization is redundant: for every function $\hat{f}$ represented by Equation (1) there exists infinitely many transformations of the parameters $\theta_{\text{NN}}^{\prime }\equiv R\left(\theta_{\text{NN}}\right)$ s.t. the transformed function $\hat{f}\left(x;\theta_{\text{NN}}^{\prime }\right)=\hat{f}\left(x;\theta_{\text{NN}}\right)$. These invariant transformations R consist of (i) permutations of the hidden units and (ii) scalings of the weights and biases of the form $w_{i}\mapsto \alpha_{i}w_{i},b_{i}\mapsto \alpha_{i}b_{i},v_{i}\mapsto \alpha_{i}^{-1}v_{i}$ for α_*i*_ ∈ ℝ_>0_ (Rolnick and Kording, 2019). The manifold of constant loss generated by the α-scaling symmetry is shown in Figure 1. The set G of such function-invariant transformations together with function composition ◦ forms a group.

**Figure 2:**
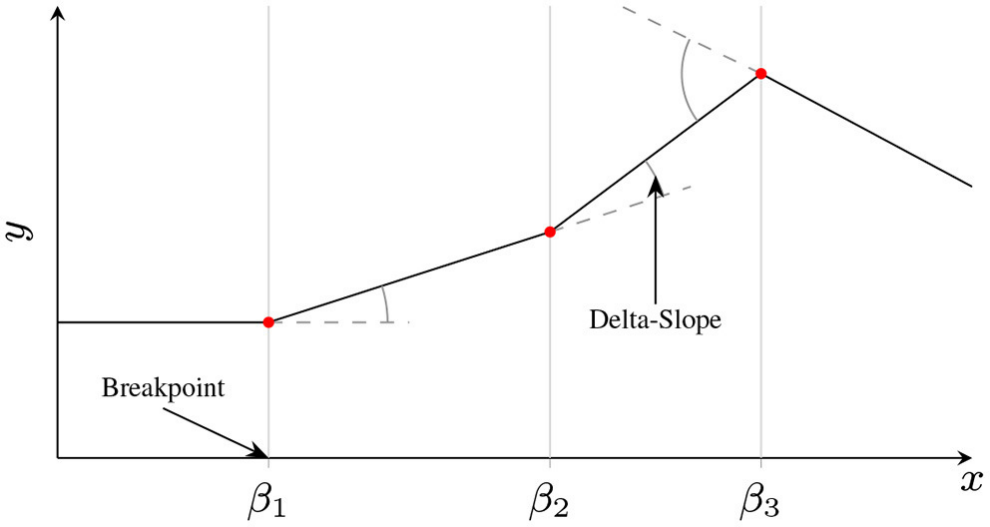
Illustration of BDSO terminology of a CPWL function. Breakpoints are where adjacent linear pieces meet, and the delta-slope determines the difference in slope between the adjacent linear pieces. Orientations determine on which side of a breakpoint the delta-slope is applied. In a shallow NN, each neuron contributes exactly one breakpoint, with an associated facing *s*_*i*_ and delta-slope μ_*i*_ – in the above example, neurons 1 and 2 are right-facing with positive delta-slope (*s*_1_ = *s*_2_ = 1, μ_1_, μ_2_ > 0), and neuron 3 is left-facing with negative delta-slope (*s*_3_ = −1, μ_3_ < 0).

We want to understand the *function* implemented by this neural net, and so we ask: How do the CPWL parameters relate to the NN parameters? We answer this by taking the quotient with respect to the above α-scaling symmetry, transforming from the NN parametrization of weights and biases to the following CPWL *spline* parametrization:


(1) $$\begin{array}{l} \hat{f}\left(x;\theta_{\text{BDSO}}\right)≜b_{0}+\overset{H}{\underset{i=1}{\sum }}\mu_{i}{\left(x-\beta_{i}\right)}_{s_{i}}, \end{array}$$

where


$${\left(x-\beta_{i}\right)}_{s_{i}}≜\left(x-\beta_{i}\right)\cdot \{\begin{array}{cc} ⟦x>\beta_{i}⟧, & \text{if }s_{i}=1\\ ⟦x<\beta_{i}⟧, & \text{if }s_{i}=-1 \end{array}$$

and the Iversen bracket ⟦*b*⟧ is 1 when the condition *b* is true, and 0 otherwise. The CPWL spline parametrization is the *B**reakpoint*, *D**elta-**S**lope*, *O**rientation (BDSO)* parametrization, $\theta_{\text{BDSO}}≜\left\{b_{0}\right\}\cup {\left\{\left(\beta_{i},\mu_{i},s_{i}\right)\right\}}_{i=1}^{H}$, where $\beta_{i}≜-\frac{b_{i}}{w_{i}}$ is (the *x*-coordinate of) the *breakpoint* (or *knot*) induced by neuron *i*, μ_*i*_ ≜ *w*_*i*_*v*_*i*_ is the *delta-slope* contribution of neuron *i*, and *s*_*i*_ ≜ sgn *w*_*i*_ ∈ {±1} is the *orientation* of β_*i*_ (left for *s*_*i*_ = −1, right for *s*_*i*_ = +1). This terminology is illustrated in Figure 2.

Considering the BDSO parametrization provides a new, useful lens with which to analyze neural nets, enabling us to reason more easily and transparently about the initialization, loss surface, and training dynamics. The benefits of this approach derive from taking the quotient with respect to the α-scaling symmetry, leading to two useful properties: (1) the loss depends on the NN parameters θ_NN_ only through the BDSO parameters (the approximating function) θ_BDSO_ i.e., ℓ(θ_NN_) = ℓ(θ_BDSO_(θ_NN_)), analogous to the concept of a minimum sufficient statistic in exponential family models; and (2) the BDSO parameters are more intuitive, allowing us to reproduce known results (Williams et al., 2019) more succinctly, and expand out to new results more easily. Much recent related work has also moved in this direction, analyzing function space (Balestriero and Baraniu, 2018; Hanin and Rolnick, 2019).

The BDSO parametrization makes clear that, fundamentally, $\hat{f}\left(x;\theta_{\text{NN}}\right)$ is represented in terms of its second derivative. Examining


(2) $$\begin{array}{l} {\hat{f}}^{\prime \prime }\left(x;\theta_{\text{BDSO}}\right)=\overset{H}{\underset{i=1}{\sum }}\mu_{i}s_{i}\delta \left(x-\beta_{i}\right) \end{array}$$

further illustrates the meaning of the BDSO parameters, especially μ_*i*_ and β_*i*_: the second derivative is zero everywhere except at *x* = β_*i*_ for some *i*, where there is a Dirac delta of magnitude μ_*i*_.

We note that the BDSO parametrization of a ReLU NN is closely related to but different than a traditional *m*th order spline parametrization ${\hat{f}}_{\text{spline}}\left(x\right)≜\overset{K}{\underset{i=1}{\sum }}\mu_{i}{\left(x-\beta_{i}\right)}_{+}^{m}+\overset{m}{\underset{j=0}{\sum }}c_{j}x^{j}$ (Reinsch, 1967). The BDSO parametrization (i) lacks the base polynomial, and (ii) has two possible breakpoint orientations *s*_*i*_ ∈ {±1} whereas the spline is canonically all right-facing. Additionally, adding in the base polynomial (for the linear case *m* = 1) into the BDSO parametrization yields a ReLU ResNet parametrization.

It is also useful to consider the set of sorted breakpoints, ${\left\{\beta_{p}\right\}}_{p=0}^{H+1}$, where −∞ ≜ β_0_ < β_1_ < ⋯ < β_*p*_ < ⋯ < β_*H*+1_ ≜ ∞, which induces two partitions: (i) a partition of the *domain* X into intervals $X_{p}≜\left[\beta_{p},\beta_{p+1}\right)$; and (ii) a partition of the *training data* $D_{x}≜{\left\{x_{n}\right\}}_{n=1}^{N}$ into pieces $\pi_{p}≜X_{p}\cap D_{x}$, so that $D_{x}=\cup_{p=0}^{H+1}\pi_{p}$. Note that sorting the breakpoints (and hence the neurons) removes the permutation symmetry present in the standard NN parametrization.

Finally, we introduce some notation relevant to the training of the network: let $\hat{\epsilon }\in {\mathbb{R}}^{N\times 1}$ denote the vector of *residuals* $\text{y}-\hat{\text{y}}$, i.e., ${\hat{\epsilon }}_{n}=f\left(x_{n}\right)-\hat{f}\left(x_{n}\right)$, let $1_{i}\in {\mathbb{R}}^{N\times 1}$ denote the *activation pattern* of neuron *i* over all *N* inputs, $1_{i,n}=⟦w_{i}x_{n}+b_{i}>0⟧$, and let ϵ^i≜ϵ^⊙1i∈ℝN×1 and ${\text{x}}_{i}≜\text{x}⊙1_{i}\in {\mathbb{R}}^{N\times 1}$ denote the *relevant* residuals and inputs for neuron *i*, respectively. In words, relevant residuals and inputs for neuron *i* are those which are not zeroed out by the ReLU activation.

#### 2.1.1. Basis Expansion, Infinite Width Limit

The expansion in Equation 2 can be further understood by viewing it as a basis expansion. Specifically, we can view the BDSO framework as a basis expansion of ${\hat{f}}^{\prime \prime }$ in a basis of Dirac delta functions, or, from Equation 1 as a basis expansion of $\hat{f}$ in a basis of functions (*x* − β_*i*_)_*s*_*i*__. The form of these basis functions is fixed as the ReLU activation—analogous to the mother basis function in wavelets (Rao, 2002)—but can be translated via changes in β_*i*_, reflected by *s*_*i*_, or re-weighted via changes in μ_*i*_.

Also, note that if the breakpoints β_*i*_ are fixed, and only the delta-slope parameters μ_*i*_ are optimized, then we effectively have a (kernel) linear regression model. Thus, the power of neural network learning lies in the ability to translate/orient these basis functions in order to better model the data. Intuitively, in a good fit, the breakpoints β_*i*_ will tend to congregate near areas of high curvature in the ground truth function, i.e., |*f*″(*x*)| ≫ 0, so that the sum-of-Diracs ${\hat{f}}^{\prime \prime }\left(x;\theta_{\text{BDSO}}\right)$ better approximates *f*″(*x*).^1^

Consider the infinite width limit *H* → ∞, rewriting Equation 2 as


(3) $$\begin{array}{l} {\hat{f}}^{\prime \prime }\left(x;\theta_{\text{BDSO}}\right)=\int c\left(\beta \right)\delta \left(x-\beta \right)\text{d}\beta \\ \;=\int p_{\beta }\left(\beta \right)\frac{c\left(\beta \right)}{p_{\beta }\left(\beta \right)}\delta \left(x-\beta \right)\text{d}\beta \\ \;={𝔼}_{\beta }\left[\frac{c\left(\beta \right)}{p_{\beta }\left(\beta \right)}\delta \left(x-\beta \right)\right]. \end{array}$$

Using the Law of Large Numbers, ${𝔼}_{\beta }\left[\frac{c\left(\beta \right)}{p_{\beta }\left(\beta \right)}\delta \left(x-\beta \right)\right]=\frac{1}{H}\sum_{i}\frac{c\left(\beta_{i}\right)}{p_{\beta }\left(\beta_{i}\right)}\delta \left(x-\beta_{i}\right)+O\left(1/H\right)$ and so we may write μ_*i*_*s*_*i*_ ≜ *c*(β_*i*_)/*Hp*_β_(β_*i*_). Then, from Equation 3, we can expand this as $\mu_{i}s_{i}={\hat{f}}^{\prime \prime }\left(\beta_{i};\theta_{\text{BDSO}}\right)/Hp_{\beta }\left(\beta_{i}\right)$.

### 2.2. Random Initialization in Function Space

We now study the random initializations commonly used in deep learning in function space. These include the independent Gaussian initialization, with $b_{i}\sim N\left(0,\sigma_{b}\right)$, $w_{i}\sim N\left(0,\sigma_{w}\right)$, $v_{i}\sim N\left(0,\sigma_{v}\right)$, and independent uniform initialization, with $b_{i}\sim U\left[-a_{b},a_{b}\right]$, $w_{i}\sim U\left[-a_{w},a_{w}\right]$, $v_{i}\sim U\left[-a_{v},a_{v}\right]$. We find that common initializations result in flat functions, becoming flatter with increasing width.

**Theorem 1**. *Consider a fully connected ReLU neural net with scalar input and output, and a single hidden layer of width* *H*. *Let the weights and biases be initialized randomly according to a zero-mean Gaussian or Uniform distribution. Then the induced distributions of the function space parameters (breakpoints β, delta-slopes μ) are as follows*:

*(a) Under an independent Gaussian initialization*,
  $$p_{\beta ,\mu }\left(\beta_{i},\mu_{i}\right)=\frac{1}{2\pi \sigma_{v}\sqrt{\sigma_{b}^{2}+\sigma_{w}^{2}\beta_{i}^{2}}}\exp\left[-\frac{|\mu_{i}|\sqrt{\sigma_{b}^{2}+\sigma_{w}^{2}\beta_{i}^{2}}}{\sigma_{b}\sigma_{v}\sigma_{w}}\right]$$
*(b) Under an independent Uniform initialization*,
  $$\begin{array}{l} p_{\beta ,\mu }\left(\beta_{i},\mu_{i}\right)=\frac{⟦|\mu_{i}|\leq \min\left\{\frac{a_{b}a_{v}}{\left|\beta_{i}\right|},a_{w},a_{v}\right\}⟧}{4a_{b}a_{w}a_{v}}\\ \;\left(\min\left\{\frac{a_{b}}{\left|\beta_{i}\right|},a_{w}\right\}-\frac{\left|\mu_{i}\right|}{a_{v}}\right) \end{array}$$

Using this result, we can immediately derive marginal and conditional distributions for the breakpoints and delta-slopes.

**Corollary 1**. *Consider the same setting as Theorem 1*.

*(a) In the case of an independent Gaussian initialization*,
  $$\begin{array}{l} \;p_{\beta }\left(\beta_{i}\right)=\mathrm{Cauchy}\left(\beta_{i};0,\frac{\sigma_{b}}{\sigma_{w}}\right)\\ \;p_{\mu }\left(\mu_{i}\right)=\frac{G_{0,2}^{2,0}\left(\frac{\mu_{i}^{2}}{4\sigma_{v}^{2}\sigma_{w}^{2}}|\begin{array}{c} 0,0 \end{array}\right)}{2\pi \sigma_{v}\sigma_{w}}=\frac{K_{0}\left(\frac{\left|\mu_{i}\right|}{\sigma_{v}\sigma_{w}}\right)}{\pi \sigma_{v}\sigma_{w}}\\ p_{\mu |\beta }\left(\mu_{i}|\beta_{i}\right)=\mathrm{Laplace}\left(\mu_{i};0,\frac{\sigma_{b}\sigma_{v}\sigma_{w}}{\sqrt{\sigma_{b}^{2}+\sigma_{w}^{2}\beta_{i}^{2}}}\right) \end{array}$$
  *where* $G_{pq}^{nm}\left(\cdot |\cdot \right)$ *is the Meijer G-function and* *K*_ν_(·) *is the modified Bessel function of the second kind*.
*(b) In the case of an independent Uniform initialization*,
  $$\begin{array}{l} \;p_{\beta }\left(\beta_{i}\right)=\frac{1}{4a_{b}a_{w}}{\left(\min\left\{\frac{a_{b}}{\left|\beta_{i}\right|},a_{w}\right\}\right)}^{2}\\ \;p_{\mu }\left(\mu_{i}\right)=\frac{⟦-a_{w}a_{v}\leq \mu_{i}\leq a_{w}a_{v}⟧}{2a_{w}a_{v}}\log\frac{a_{w}a_{v}}{\left|\mu_{i}\right|}\\ p_{\mu |\beta }\left(\mu_{i}|\beta_{i}\right)=\mathrm{Tri}\left(\mu_{i};a_{v}\min\left\{a_{b}/|\beta_{i}|,a_{w}\right\}\right) \end{array}$$
  *where* Tri(·;*a*) *is the symmetric triangular distribution with base* [−*a, a*] *and mode* 0.

#### 2.2.1. Implications

Corollary 1 implies that the breakpoint density drops quickly away from the origin for common initializations. As breakpoints are necessary to fit curvature, if the ground truth function *f*(*x*) has significant curvature far from the origin, then it may be far more difficult to fit. We show that this is indeed the case by training a shallow ReLU NN with an initialization that does not match the underlying curvature, with training becoming easier if the initial breakpoint distribution better matches the function curvature. This suggests that a data-dependent initialization, with more breakpoints near areas of high curvature, could potentially be faster and easier to train. Note that this simple curvature-based interpretation is made possible by our symmetry-quotiented reparametrization.

Next, we consider the typical Gaussian He (He et al., 2015) or Glorot (Glorot and Bengio, 2010) initializations. In the He initialization, we have $\sigma_{w}=\sqrt{2}$, $\sigma_{v}=\sqrt{2/H}$. In the Glorot initialization, we have $\sigma_{w}=\sigma_{v}=\sqrt{2/\left(H+1\right)}$. We wish to consider their effect on the smoothness of the initial function approximation. From here on, we measure the smoothness using the *roughness* of $\hat{f}$, $\rho ≜\int |{\hat{f}}^{\prime \prime }\left(x;\theta_{\text{BDSO}}\right){|}^{2}\text{d}x=\sum_{i}\mu_{i}^{2}={\left\|\mu \right\|}_{2}^{2}$, where lower roughness indicates a smoother approximation.

**Theorem 2**. *Consider the initial roughness ρ*_0_ *under a Gaussian initialization. In the He initialization, we have that the tail probability is given by*


$$\mathbb{P}\left[\rho_{0}-𝔼\left[\rho_{0}\right]\geq \lambda \right]\leq \frac{1}{1+\frac{\lambda^{2}H}{128}},$$

*where* 𝔼[ρ_0_] = 4. *In the Glorot initialization, we have that the tail probability is given by*


$$\mathbb{P}\left[\rho_{0}-𝔼\left[\rho_{0}\right]\geq \lambda \right]\leq \frac{1}{1+\frac{\lambda^{2}{\left(H+1\right)}^{4}}{128H}},$$

*where* $𝔼\left[\rho_{0}\right]=\frac{4H}{{\left(H+1\right)}^{2}}=O\left(\frac{1}{H}\right)$.

Thus, as the width *H* increases, the distribution of the roughness of the initial function ${\hat{f}}_{0}$ gets tighter around its mean. In the case of the He initialization, this mean is constant; in the Glorot initialization, it decreases with *H*. In either case, for reasonable widths, the initial roughness is small with high probability, corresponding to small initial delta-slopes. This smoothness has implications for the implicit regularization phenomenon observed in recent work (Neyshabur et al., 2018), and studied later in section 2.6, in particular, Theorem 7.

*Related Work*. Several recent works analyze the random initialization in deep networks. However, there are two main differences; first, they focus on the infinite width case (Neal, 1994; Lee et al., 2017; Jacot et al., 2018; Savarese et al., 2019) and can thus use the Central Limit Theorem (CLT), whereas we focus on finite width case and cannot use the CLT, thus requiring nontrivial mathematical machinery (see Supplement for detailed proofs). Second, they focus on the activations as a function of input whereas we also compute the joint densities of the BDSO parameters i.e., breakpoints and delta-slopes. The latter is particularly important for understanding the non-uniform density of breakpoints away from the origin as noted above. The most closely related work is Steinwart (2019), which considers only the breakpoints, and suggests a new initialization with uniform breakpoint distribution.

### 2.3. Loss Surface in the Spline Parametrization

**Figure 3:**
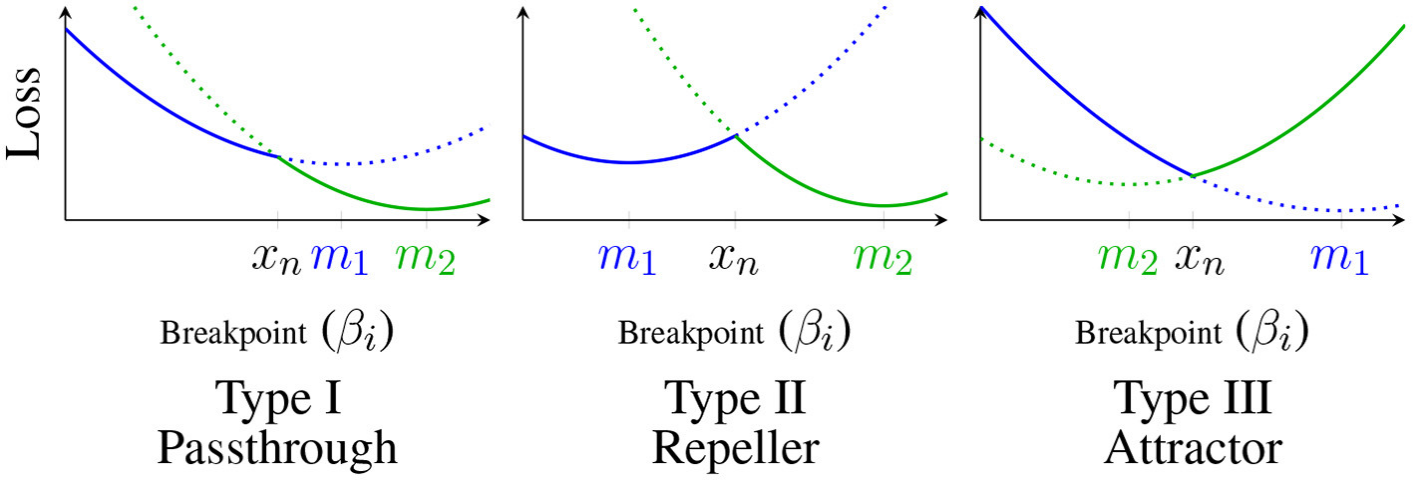
Classification of critical points: *p*_1_(β_*i*_) shown in blue, *p*_2_(β_*i*_) shown in green; solid lines represent the 1 dimensional loss slice $\tilde{\ell }\left(\beta_{i};\beta_{-i},\mu ,\text{s}\right)$, dotted lines represent the extension of *p*_*k*_(β_*i*_) past *x*_*n*_.

We now consider the squared loss $\frac{1}{2}\overset{N}{\underset{n=1}{\sum }}{\left(\hat{f}\left(x_{n};\theta \right)-y_{n}\right)}^{2}$ as a function of either the NN parameters ℓ(θ_NN_) or the BDSO parameters $\tilde{\ell }\left(\theta_{\text{BDSO}}\right)$. We begin with the symmetry-quotiented case:

**Theorem 3**. *The loss function* $\tilde{\ell }\left(\theta_{\text{BDSO}}=\left(\beta ,\mu ,\text{s}\right)\right)$ *is a continuous piecewise quadratic (CPWQ) spline. Furthermore, consider the evolution of the loss as we vary β*_*i*_ *along the* *x* *axis; this 1-dimensional slice* $\tilde{\ell }\left(\beta_{i};\beta_{-i},\mu ,\text{s}\right)$ *is also a CPWQ spline in β*_*i*_ *with knots at datapoints* ${\left\{x_{n}\right\}}_{n=1}^{N}$. *Let* *p*_1_(β_*i*_) *(resp.* *p*_2_(β_*i*_)*) be the quadratic function equal to* $\tilde{\ell }\left(\beta_{i};\beta_{-i},\mu ,\text{s}\right)$ *for β*_*i*_ ∈ [*x*_*n*−1_, *x*_*n*_] *(resp.* [*x*_*n*_, *x*_*n*+1_)*], which both have positive curvature, and let* *m*_*j*_ ≜ arg min *p*_*j*_(β_*i*_). *Then, with measure 1, the knots* *x*_*n*_ *fall into one of three types as shown in* Figure 3:

- *(Type I, Passthrough)* *m*_1_, *m*_2_ < *x*_*n*_, *or* *x*_*n*_ < *m*_1_, *m*_2_,
- *(Type II, Repeller)* *m*_1_ < *x*_*n*_ < *m*_2_
- *(Type III, Attractor)* *m*_2_ < *x*_*n*_ < *m*_1_.

We call Type I knots Passthrough knots because gradient flow starting from the higher side will simply pass through the knot. Similarly, we call Type II knots Repellers and Type III knots Attractors because β_*i*_ will be repelled by or attracted to the knot during gradient flow.

We now return to the loss ℓ(θ_NN_) under the NN parametrization, and consider varying (*w*_*i*_, *v*_*i*_, *b*_*i*_) such that the corresponding β_*i*_ changes but μ_*i*_ and *s*_*i*_ stay the same. Due to the α-scaling symmetry, this can be implemented in two distinct ways: (i) (*w*_*i*_, *v*_*i*_, *b*_*i*_) ↦ (*w*_*i*_, *v*_*i*_, *b*_*i*_ + δ) or (ii) (*w*_*i*_, *v*_*i*_, *b*_*i*_) ↦ ((1+δ)*w*_*i*_, *v*_*i*_/(1+δ), *b*_*i*_). Version (ii) can be implemented by applying version (i) followed by the transformation (*w*_*i*_, *v*_*i*_, *b*_*i*_) ↦ (α*w*_*i*_, *v*_*i*_/α, α*b*_*i*_) for the appropriate α, which leaves the loss invariant. In fact, there is a continuum of transformations which could be achieved by version (i) followed by an arbitrary α-transformation. Thus, consider the 1-dimensional slice $\ell \left(b_{i};\text{w},\text{v},{\text{b}}_{-i}\right)$, which is equal to $\tilde{\ell }\left(-b_{i}/w_{i};\beta_{-i},\mu ,\text{s}\right)$, i.e., a horizontally reflected and scaled version of $\tilde{\ell }\left(\beta_{i};\beta_{-i};\mu ,\text{s}\right)$. The 1-dimensional “slice” corresponding to version (ii) will similarly be a stretched version of $\tilde{\ell }\left(\beta_{i};\beta_{-i};\mu ,\text{s}\right)$, with the caveat that this “slice” is along a hyperbola in the (*v*_*i*_, *w*_*i*_)-plane. Noting that any changes in (*w*_*i*_, *v*_*i*_, *b*_*i*_) that implement the same change in β_*i*_ change the loss in the same way, let ℓ(β_*i*_; θ_NN_\β_*i*_) denote the equivalence class of 1-dimensional slices of ℓ(θ_NN_). This gives the following corollary:

**Corollary 2**. *The 1-dimensional slices* ℓ(β_*i*_; θ_*NN*_\β_*i*_) *of* ℓ(θ_*NN*_) *that implement a change only in* β_*i*_ *are CPWQ with knots as in Theorem 3*.

#### 2.3.1. Critical Points of the Loss Surface

In addition to the fixed points associated with attractor datapoints, there are also critical points that lie in the quadratic regions, i.e., local (and global) minima which correspond to the minima of some parabolic piece for each neuron. We characterize these critical points here.

**Theorem 4**. *Consider some arbitrary* $\theta_{\text{NN}}^{*}$ *such that there exists at least one neuron that is active on all data. Then*, $\theta_{\text{NN}}^{*}$ *is a critical point of* $\tilde{\ell }\left(\cdot \right)$ *if and only if for all domain partitions* $X_{p}$, *the restriction* $\hat{f}\left(\cdot ;\theta_{\text{NN}}^{*}\right){|}_{X_{p}}$ *is an Ordinary Least Squares (OLS) fit of the training data contained in π*_*p*_.


(4) $$\begin{array}{l} H_{\ell }\left(\theta \right)=\left(\begin{array}{ccccc} \ddots \\ \langle v_{j}{\text{x}}_{j},v_{i}{\text{x}}_{i}\rangle & \langle v_{j}{\text{x}}_{j},w_{i}{\text{x}}_{i}+b_{i}1_{i}\rangle & \langle v_{j}{\text{x}}_{j},v_{i}1_{i}\rangle & \cdots & \langle v_{j}{\text{x}}_{j},1\rangle \\ \langle w_{j}{\text{x}}_{j}+b_{j}1_{j},v_{i}{\text{x}}_{i}\rangle & \langle w_{j}{\text{x}}_{j}+b_{j}1_{j},w_{i}{\text{x}}_{i}+b_{i}1_{i}\rangle & \langle w_{j}{\text{x}}_{j}+b_{j}1_{j},v_{i}1_{i}\rangle & \cdots & \langle w_{j}{\text{x}}_{j}+b_{j}1_{j},1\rangle \\ \langle v_{j}1_{j},v_{i}{\text{x}}_{i}\rangle & \langle v_{j}1_{j},w_{i}{\text{x}}_{i}+b_{i}1_{i}\rangle & \langle v_{j}1_{j},v_{i}1_{i}\rangle & \cdots & \langle v_{j}1_{j},1\rangle \\ \vdots & & & \ddots & \vdots \\ \langle 1,v_{i}{\text{x}}_{i}\rangle & \langle 1,w_{i}{\text{x}}_{i}+b_{i}1_{i}\rangle & \langle 1,v_{i}1_{i}\rangle & \cdots & \langle 1,1\rangle \end{array}\right) \end{array}$$

An open question is how many such critical points exist. A starting point is to consider that there are *C*(*N*+*H, H*)≜(*N*+*H*)!/*N*!*H*! possible partitions of the data. Not every such partition will admit a piecewise-OLS solution which is also continuous, and it is difficult to analytically characterize such solutions.

**Figure 4:**
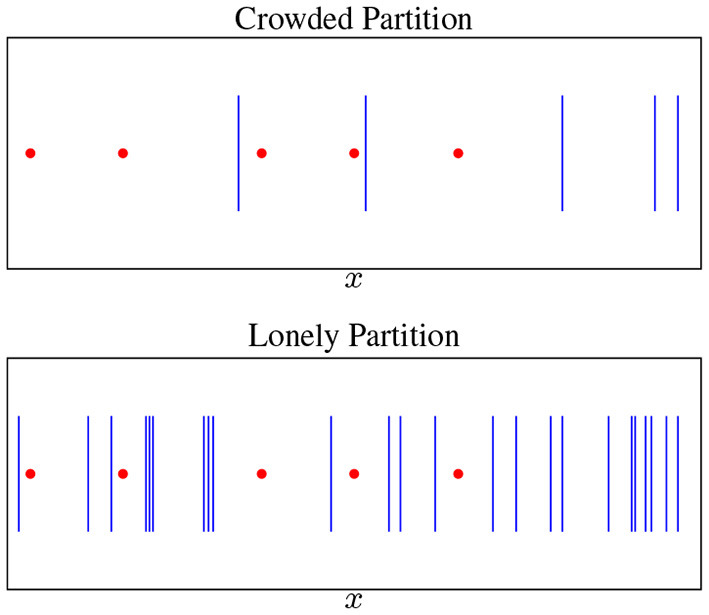
Breakpoints (blue bars) vs. datapoints (red points). A lonely partition is when every datapoint is isolated—overparametrization makes this increasingly likely.

Using Theorem 4, we can characterize growth of global minima in the overparametrized case. Call a partition Π *lonely* if each piece π_*p*_ ∈ Π contains at most one datapoint (see Figure 4). Then, we can prove the following results:

**Lemma 1**. *For any lonely partition* Π, *there are infinitely many parameter settings θ*_*BDSO*_ *that induce* Π *and are global minima with* $\tilde{\ell }\left(\theta_{\text{BDSO}}\right)=0$. *Furthermore, in the overparametrized regime* *H* ≥ *cN* *for some constant* *c* ≥ 1, *the total number of lonely partitions, and thus a lower bound on the total number of* *global* *minima of* $\tilde{\ell }$ is (H+1N) = *O*(*N*^*cN*^).

**Remark 1**. *Suppose that the *H* breakpoints are uniformly spaced and that the* *N* *datapoints are uniformly distributed within the region of breakpoints. Then in the overparametrized regime* *H* ≥ *dN*^2^ *for some constant* *d* ≥ 1, *the induced partition* Π *is lonely with high probability* 1 − *e*^−*N*^2^/(*H*+1)^ = 1 − *e*^−1/*d*^.

Thus, with only *O*(*N*^2^) hidden units, we can almost guarantee a lonely partition at initialization. This makes optimization easier but is not sufficient to guarantee that learning will converge to a global minimum, as breakpoint dynamics could change the partition to crowded (see section 2.5). Note how simple and transparent the spline-based explanation is for why overparametrization makes optimization easier. For brevity, we include experiments testing our theory of loneliness in Figure 14 in Appendix.

### 2.4. The Gradient and Hessian of the Loss ℓ(θ_NN_)

Previous works has revealed many key insights into the Hessian of the loss surface of neural networks. It has long been empirically known that GD tends to work surprisingly well, and that most or all local minima are actually near-optimal (Nguyen and Hein, 2017), although this is known to depend on the overparametrization ratio and the data distribution (Pennington and Bahri, 2017; Granziol et al., 2019). Flatter local minima have been shown to be connected to better generalization (Sankar et al., 2020), but the areas around critical points may be unusually flat, with the bulk of Hessian eigenvalues near 0, and only a few larger outliers (Ghorbani et al., 2019), meaning that many local minima are actually connected within a single basin of attraction (Sagun et al., 2017). Our theory can shed new light on many of these phenomena:

The gradient of ℓ(θ_NN_) can be expressed as


(5) ∂ℓ∂b0=-〈ϵ^,1〉∂ℓ∂wi=-〈ϵ^i,vixi〉∂ℓ∂vi=-〈ϵ^i,wixi+bi1i〉∂ℓ∂bi=-〈ϵ^i,vi1i〉

From this, we can derive expressions for the Hessian of the loss ℓ(θ_NN_) at any parameter θ_NN_.

**Theorem 5**. Let θ^*^ *be a critical point of ℓ*(·) *such that* $\beta_{i}^{*}\left(\theta^{*}\right)\neq x_{n}$ *for all* *i* ∈ [*H*] *and for all* *n* ∈ [*N*]. *Then the Hessian* ${\text{H}}_{\ell }\left(\theta^{*}\right)$ *is the positive semi-definite Gram matrix of the set of* 3*H* + 1 *vectors*


$$B≜{\left\{v_{i}{\text{x}}_{i},w_{i}{\text{x}}_{i}+b_{i}1_{i},v_{i}1_{i}\right\}}_{i=1}^{H}\cup \left\{1\right\},$$

*as shown in eq*. (4). *Thus*, ${\text{H}}_{\ell }\left(\theta^{*}\right)$ *is positive definite iff the vectors of this set are linearly independent*.

**Remark 2**. *The zero eigenvalues of* ${\text{H}}_{\ell }\left(\theta^{*}\right)$ *correspond to*

**Figure 5:**
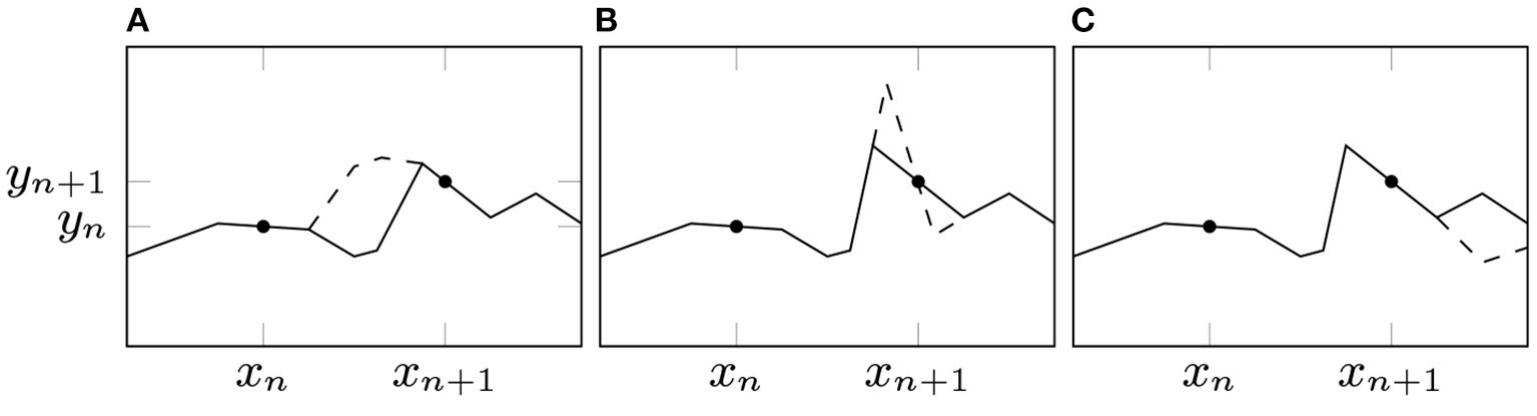
Examples of functional changes that leave the loss invariant: **(A)** when there are multiple breakpoints with the same facing in the interval (*x*_*n*_, *x*_*n*+1_), they can change in ways that cancel out, leaving the function unchanged on the data; **(B)** if two breakpoints on either side of a datapoint (with no other data in between) have the same facing, they may “rotate” the portion of the function between them around *x*_*n*_; **(C)** any neuron outside of the data range and facing away (which therefore has no active data) is completely unconstrained by the loss until β_*i*_ enters the data range.

1. ***α-scaling:*** *For each neuron* *i*, *the scaling transformation* $\left(w_{i},b_{i},v_{i}\right)\mapsto \left(\alpha_{i}w_{i},\alpha_{i}b_{i},\alpha_{i}^{-1}v_{i}\right)$ *leaves the approximating function* $\hat{f}\left(x\right)$ (*and thus the training loss*) *invariant, thus generating a 1-dimensional hyperbolic manifold of constant loss; thus, the tangent vector of this manifold will be in the null space of* ${\text{H}}_{\ell }\left(\theta \right)$ *for all θ. This manifold is depicted in* ***Figure 1***.
2. ***0 singularities:*** *For each neuron* *i*, *if either of* *w*_*i*_ *or* *v*_*i*_ *are 0*:
  - *if* *v*_*i*_ = 0, *then μ*_*i*_ = 0, *so that changes to the value of β*_*i*_ (*via either* *b*_*i*_ *or* *w*_*i*_) *do not change* $\hat{f}\left(x\right)$, *thus contributing two 0 eigenvalues*
  - *if* *w*_*i*_ = 0, *then μ*_*i*_ = 0 *and β*_*i*_ = ±∞, *so any changes to* *b*_*i*_ *or* *v*_*i*_ *do not change* $\hat{f}\left(x\right)$, *thus contributing two 0 eigenvalues*
3. ***Functional changes:*** *Any functional change that does not change the value of* $\hat{f}\left(x\right)$ *at the training data will leave the loss unchanged. In the sufficiently overparametrized regime, there are many ways to make such changes. Some examples are shown in* Figure 5.

*The first two types are artifacts of the NN parametrization, caused by invariance to the α-scaling symmetry*.

Additionally, we note that these are all also zero eigenvalues for non-critical points, but that there are other zero eigenvalues for non-critical points. While ${\text{H}}_{\ell }\left(\theta^{*}\right)$ does not depend on ϵ at the above critical points, at other points, ϵ terms can “cancel out” the corresponding term in eq. (4), yielding additional zero eigenvalues (as well as negative eigenvalues).

From this, we note that for any critical point θ^*^, there is a large connected sub-manifold of parameter space of constant loss. Moving along this manifold consists of continuously deforming $\hat{f}\left(x;\theta_{\text{NN}}^{*}\right)$ such that the output values at datapoints are left invariant or moving along the α-scaling manifolds which leave $\hat{f}\left(x;\theta_{\text{NN}}^{*}\right)$ invariant. The 0 singularities lie at the intersection of every α-scaling manifold for neuron *i*, corresponding to the α = 0 and α = ∞ limits.

Consider the μ-only Hessian under the BDSO parametrization, $\frac{d2\ell }{d\mu 2}$, and rewrite our network as $\hat{\text{y}}=\Phi \left(\text{x};\beta \right)\mu$, where Φ is the *N* × *H* feature matrix of activations (or basis functions) (*x*_*n*_ − β_*i*_)_*s*_*i*__, which is constant with respect to μ. Then, we have $\frac{d2\ell }{d\mu 2}=\Phi^{⊤}\Phi$. Applying an SVD decomposition $\Phi =USV^{⊤}$, then $\Phi^{⊤}\Phi =VS^{2}V^{⊤}$, such that the eigenvalues of the Hessian will be the singular values of Φ squared. Thus, we can decompose the vector of delta-slopes $\mu =\mu_{r}+\mu_{n}$ where $\mu_{r}$ is in the range of Φ while $\mu_{n}$ is in the nullspace. Thus, $\hat{\text{y}}=\Phi \left(\text{x};\beta \right)\mu_{r}+\Phi \left(\text{x};\beta \right)\mu_{n}=\Phi \left(\text{x};\beta \right)\mu_{r}$, and the gradient with respect to μ is always constrained to the linear subspace $\mu_{r}$.

**Figure 6:**
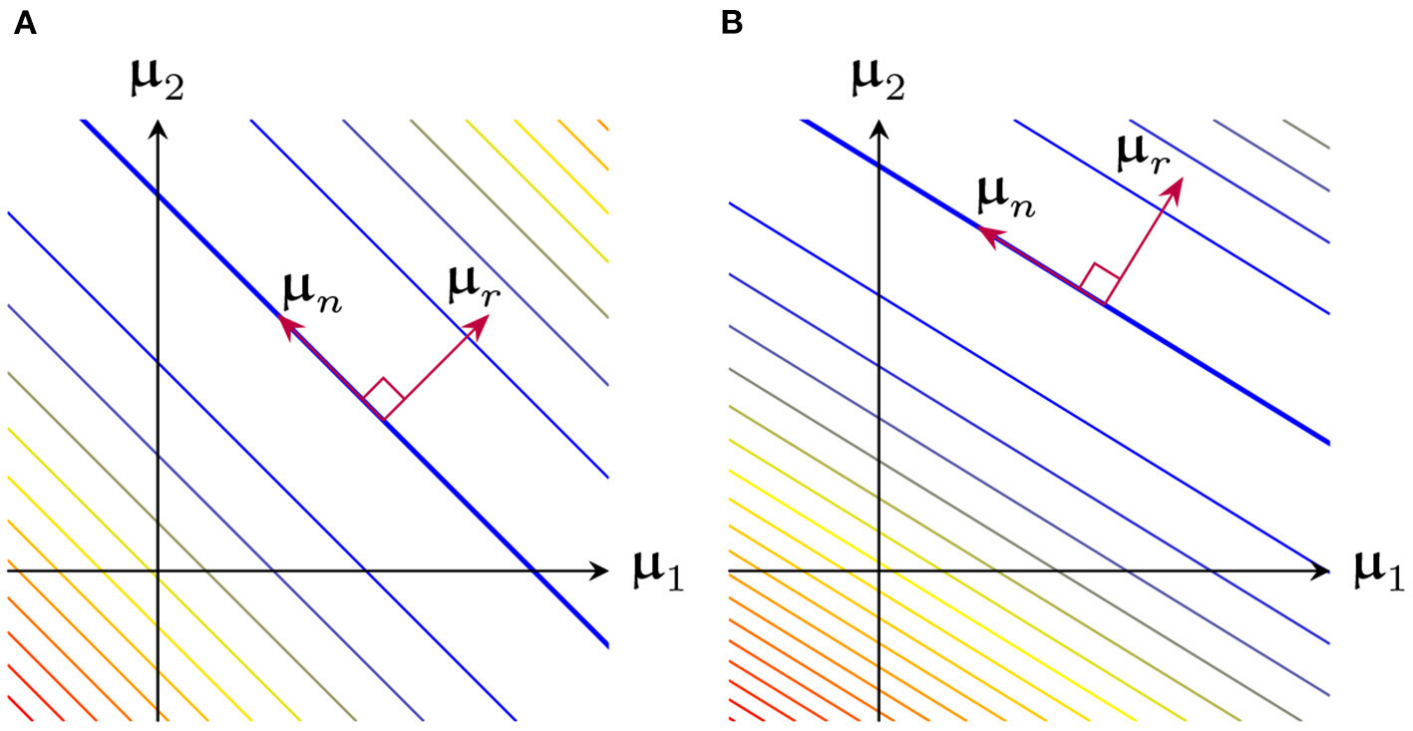
2-dimensional representation of the loss surface as a function of μ, broken down into a loss-sensitive range and loss-independent nullspace **(A)** Example for fixed β **(B)** Same system as before, except β was varied, resulting in modified μ range and nullspace.

Figure 6A shows a 2-dimensional representation of the loss surface, which is quadratic along $\mu_{r}$, and constant along $\mu_{n}$. Figure 6B illustrates the effect of varying β, which changes Φ(x;β) and hence $\mu_{r}$ and $\mu_{n}$.

#### 2.4.1. The Flatness of the Hessian

An important question is: How does overparametrization impact (the spectrum of) the Hessian? The symmetry-quotiented spline parametrization enables us to lower bound the number of zero eigenvalues of the Hessian [e.g., the “flatness” (Li et al., 2018)] as follows.

**Corollary 3**. Let θ^*^ *be a critical point of* ℓ(·) *such that its data partition is lonely, and either at least one neuron is active on all data or there is at least one pair of oppositely-faced neurons in the same data gap, so that* x∈span(B). *Then*, ℓ(θ^*^) = 0 *and* ${\text{H}}_{\ell }\left(\theta^{*}\right)$ *has exactly* *N* *non-zero eigenvalues, and thus* 3*H* + 1 − *N* *zero eigenvalues*.

Intuitively, as overparametrization *H*/*N* increases, the number of neurons with shared activation patterns increases, which in turn means many redundant breakpoints between each pair of datapoints, which increases the number of flat directions (zero eigenvalues) of the Hessian. Additionally, each neuron is guaranteed one degree of freedom in the form of the α-scaling symmetry, leading to *H* intrinsic zero eigenvalues of the Hessian.

### 2.5. Gradient Flow Dynamics for Spline Parameters

**Theorem 6**. *For a one hidden layer univariate ReLU network trained with gradient descent with respect to the neural network parameters* $\theta_{\text{NN}}={\left\{\left(w_{i},b_{i},v_{i}\right)\right\}}_{i=1}^{H}$, *the gradient flow dynamics of the function space parameters* $\theta_{\text{BDSO}}={\left\{\left(\beta_{i},\mu_{i}\right)\right\}}_{i=1}^{H}$ *are governed by the following laws*:


(6) βi˙=vi(t)wi(t)[〈ϵ^i(t),1〉︸net relevant residual+βi(t)〈ϵ^i(t),x〉︸correlation]

(7) μi˙=wi2(t)[-(1+(vi(t)wi(t))2)〈ϵ^i(t),x〉+βi(t)〈ϵ^i(t),1〉]

Here *i* ∈ [*H*] indexes all hidden neurons and the initial conditions β_*i*_(0), μ_*i*_(0)∀*i* ∈ [*H*] must be specified by the initialization used (see Appendix B.8 for derivation).

#### 2.5.1. Impact of Init Scale α

As mentioned previously, the standard NN parametrization has symmetries such that the function $\hat{f}$ and the loss is invariant to α-scaling transformations of the NN parameters (section 2.1). However, such scalings do have a large effect on the *initial* NN gradients; specifically, applying the α-scaling transformation in eqs. (6) and (7), the *initial* learning rate for β_*i*_ scales as $1/\alpha_{i}^{2}$, while that of μ_*i*_ scales approximately as $\alpha_{i}^{2}$. Changing α at initialization has a large impact on the final learned solution. In Section 2.6 we will show how α determines the kind of (implicit) regularization seen in NN training (Woodworth et al., 2020).

#### 2.5.2. Breakpoint Dynamics

Next, we wish to build some intuition of the conditions that lead to the different knot types and the effect the types have on training dynamics and implicit regularization. First, we rewrite eq. (6) as


βi˙=vi(t)wi(t)〈ϵ^i(t),1i+βi(t)xi〉,

and note that the dot product is a cumulative sum of weighted residuals (summing from the furthest datapoint on the active side of neuron *i* and moving toward β_*i*_). Next, let $A_{i}=\left\{n|1_{i,n}=1\right\}$ denote the set of data indices which are active for neuron *i*, and $X_{i}$ be the active half-space of neuron *i* [i.e., (−∞, β_*i*_) or (β_*i*_, ∞)]. If we view ϵ^i(t) as ϵ^(βi,t)≜∑n∈Aiϵ^nδ(βi-xn), we can rewrite the dot product as an integral and write $\dot{\beta_{i}}$ as a function ϱ_*i*_(β, *t*):


ϱi(β,t)=vi(t)wi(t)∫Xiϵ^(x,t)(1+βx)dx                ≜vi(t)wi(t)ψsi(β,t)

Finally, note that ψ_+_(β, *t*) = ψ_−_(∞, *t*)−ψ_−_(β, *t*), i.e., ψ_+_(·, ·) is just ψ_−_(·, ·) reflected across the (finite) total integral; accordingly, we drop the subscript and refer to ψ(·, ·) from now on.

Then, to identify Repeller and Attractor knots, we are interested in the datapoints β_*i*_ = *x*_*n*_ where ${\dot{\beta }}_{i}$ changes sign, i.e., we are interested in the zero-crossings of ψ(·, *t*). Very roughly, at earlier stages of learning, large regions of the input space have high residual, so that many neurons get large, similar updates, leading to broad, smooth changes to $\hat{f}\left(x;\theta_{\text{NN}}\right)$ and hence broad, smooth changes to ϵ^(x,t) and ψ(β, *t*). Applying such a change to ψ(β, *t*) will have the effect of “centering” ψ(β, *t*) such that it has an average value of 0 over large regions. Because the changes are broad at first, the steeper regions of ψ(β, *t*) will retain most of their steepness. Together with the “centering,” this leads to the conclusion that the local extrema of $\frac{\partial \psi \left(\beta ,t\right)}{\partial \beta }$ will be near zero-crossings of ψ(β, *t*).


∂ψ(β,t)∂β=ϵ^(β,t)(1+β2)+∫-∞βϵ^(x,t)xdx

Due to the broad centering effect, the second term ≈ 0,


≈ϵ^(β,t)(1+β2)

Therefore, the local extrema of ϵ^(β,t) will be local extrema of $\frac{\partial \psi \left(\beta ,t\right)}{\partial \beta }$ and hence near zero-crossings of ψ(β, *t*).

Note that the above analysis shows that ${\dot{\beta }}_{i}$ and hence the classification of each datapoint is determined by the same ψ(β, *t*), plus a scaling factor *v*_*i*_(*t*)/*w*_*i*_(*t*) and possibly a reflection based on sgn(*w*_*i*_), i.e., groups of neurons with the same signs of *v*_*i*_ and *w*_*i*_ will yield the same datapoint classification. If datapoint persists in being an Attractor knot for a group of nearby neurons for a long enough time, and is surrounded by Passthrough knots, then those nearby neurons will converge on the datapoint and form a cluster. This leaves the question: what conditions lead to local extrema of ϵ^(β,t) that persist long enough for this behavior?

**Figure 7:**
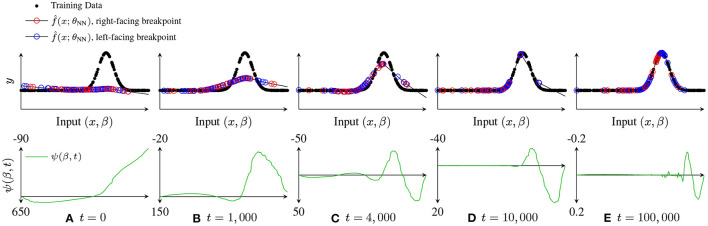
Illustration of breakpoint dynamics, showing how $\hat{f}\left(x;\theta_{\text{NN}}\right)$ and the breakpoint dynamics factor ψ(β, *t*) evolve over time. For *t* = 0, 1, 000, 4, 000, 10, 000, 100, 000. **(A)** *t* = 0, **(B)** *t* = 1,000, **(C)** *t* = 4,000, **(D)** *t* = 10,000, and **(E)** *t* = 100,000.

Empirically, we find *concentrations* of breakpoints forming near regions of high curvature or discontinuities in the training data, leading to a final fit that was close to a linear spline interpolation of the data. In particular, breakpoints migrate toward nearby curvature which is being underfit and whose inflection is the same as that breakpoint's delta-slope. These clusters can remain fixed for some time, before suddenly shifting (all breakpoints in a cluster move together away from the shared attractor *x*_*n*_) or splitting (two groups of β form two new sub-clusters that move away from *x*_*n*_ in each direction). These new movements can cause “smearing,” when the cluster loses coherence. Sometimes, this “smearing” effect is caused by the need to fit smooth curvature near a cluster: once the residual at the cluster falls below the residual of nearby curvature being underfit by the linear fit provided between clusters, the tendency is for the cluster to spread out; see Figure 7E.

As an illustrative example to explore this question, consider fitting a data set such as that shown in Figure 7. At initialization (Figure 7A), $\hat{f}\left(x;\theta_{\text{NN}}\right)$ is much flatter than *f*(*x*). After a short time of training (Figure 7B), $\hat{f}\left(x;\theta_{\text{NN}}\right)$ has matched the average value of *f*(*x*), but is still much flatter, leading to relatively broad regions where ϵ^(β,t) is large and of the same sign, with zero crossings of ψ(β, *t*) near the first two inflection points. After a little more training (Figure 7C), we see that all three inflection points correspond to zero crossings in ψ(β, *t*), and that these zero crossings have persisted long enough that clusters of breakpoints have started to form, although the leftmost inflection point is already fit well enough that ψ(β, *t*) (and hence ${\dot{\beta }}_{i}$ is quite low in the region, so that those breakpoints are unlikely to make it “all the way” to a cluster before the data is well fit. Later (Figure 7D), the right two inflection points have nearby tight clusters. However, once $\hat{f}\left(x;\theta_{\text{NN}}\right)$ reaches *f*(*x*) at these clusters, the local maxima of ϵ^(β,t) move away from the clusters, and the clusters “spread out,” as shown in the converged fit Figure 7E.

#### 2.5.3. Videos of Gradient Descent/Flow Dynamics

We have developed a BDSO spline parametrization-based visualization. For many of the experiments in this paper, the corresponding videos showing the learning dynamics are available at http://shorturl.at/tFWZ2.

### 2.6. Implicit Regularization

One of the most useful and perplexing properties of deep neural networks has been that, in contrast to other high capacity function approximators, overparametrizing a neural network does not tend to lead to excessive overfitting (Savarese et al., 2019). Where does this generalization power come from? What is the mechanism? One promising idea is that implicit regularization (IR) plays a role. IR acts as if a extra regularization term had been applied to the optimizer, despite no such regularization term being explicitly added by a designer. Instead, IR is added implicitly, brought about by some combination of architecture, initialization scheme, or optimization. Much recent work (Neyshabur et al., 2015, 2018) has argued that this IR is due to the optimization algorithm itself (i.e., SGD). Our symmetry-quotiented spline perspective makes the role of α-scaling symmetry apparent: IR depends critically on breakpoint and delta-slope learning dynamics and initialization. These results follow from section 2.5, showing that changes to the initialization scale result in dramatic changes to the relative speeds of learning dynamics for different parameters. In particular, in the kernel regime α → ∞, breakpoints move very little whereas delta-slopes move a lot, resulting in diffuse populations of breakpoints that distribute curvature. In stark contrast, in the adaptive regime α → 0 breakpoints move a lot whereas delta-slopes move very little, resulting in tight clusters of breakpoints that concentrate curvature.

#### 2.6.1. Kernel Regime

We first analyze the so-called kernel regime, inspired by Chizat et al. (2019) where it is referred to as “lazy training.” In this section, we omit the overall bias *b*_0_.

**Lemma 2**. *Consider the dynamics of gradient flow on ℓ*(·) *started from* $\theta_{\text{NN},\alpha }≜\left(\alpha {\text{w}}_{0},\alpha {\text{b}}_{0},{\text{v}}_{0}=0\right)$, *where* *w*_*i*_ ≠ 0∀*i* ∈ [*H*]. *In the limit* α → ∞, β(t) *does not change, i.e., each breakpoint location and orientation is fixed. In this case, the θ*_*NN*_ *model reduces to a (kernel) linear regression:*


(8) $$\begin{array}{l} \hat{\text{y}}=\Phi \left(\text{x};\beta \right)\mu \end{array}$$

*where* $\mu \in {\mathbb{R}}^{H}$ *are the regression weights and* **Φ**(x; **β**) ∈ ℝ^*N*×*H*^ *are the nonlinear features* ϕ_*ni*_≜(_*x*_*n*_−β_*i*_)*s*_*i*__.

Using this, we can then adapt a well known result about linear regression (see e.g., Zhang et al., 2016):

**Theorem 7**. *Let* $\mu^{*}$ be the converged μ *parameter after gradient flow on the BDSO model eq. (8) starting from some* $\mu_{0}$, *with* β *held constant. Furthermore, suppose that the model perfectly interpolates the training data* $\tilde{\ell }\left(\theta_{\text{BDSO}}\right)=0$. *Then*,


$$\mu^{*}=\underset{\mu }{arg min}{\left\|\mu -\mu_{0}\right\|}_{2}^{2}\text{ s.t. }y=\Phi \left(x;\beta \right)\mu .$$

**Figure 8:**
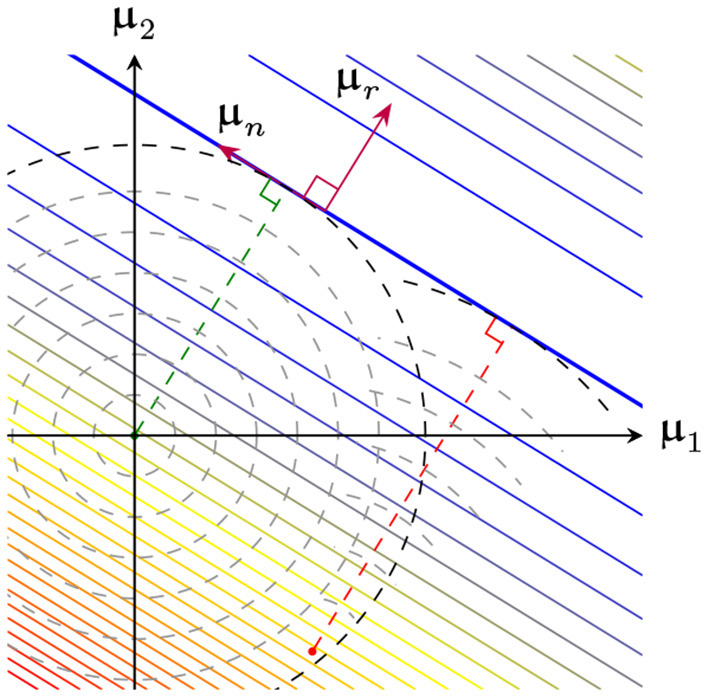
View of the loss surface as in Figure 6. GD starting from the origin (green dashed line) monotonically increases ${\left\|\mu \right\|}_{2}$ (shown as concentric circles). GD starting from some other initialization (red dashed line) minimizes ${\left\|\mu -\mu_{0}\right\|}_{2}$ instead (shown as concentric arcs).

Thus, the case where breakpoint locations and orientations are fixed, and $\mu_{0}=0$ reduces to ℓ_2_-regularized linear regression on the delta-slope parameters μ. A geometric representation of this process is shown in Figure 8. Recalling that ${\left\|\mu \right\|}_{2}^{2}$ is the roughness of $\hat{f}\left(x;\theta_{\text{BDSO}}\right)$, this result demonstrates the importance of the initialization, and in particular the initial roughness (Theorem 2): with a high-roughness initialization minimizing ${\left\|\mu -\mu_{0}\right\|}_{2}^{2}$ will yield a high-roughness final fit.

In the overparametrized regime with an initialization that yields a lonely partition, the model will reach $\tilde{\ell }\left(\theta_{\text{BDSO}}\right)=0$. If we consider the infinite-width limit, we get the following result, which is a specialization of Theorem 5 of Williams et al. (2019):

**Corollary 4**. *Consider the setting of Theorem 7, with the additional assumptions that* $\mu_{0}=0$ *and the breakpoints are uniformly spaced, and let* *H* → ∞. *Then the learned function* ${\hat{f}}_{\infty }\left(x;\mu^{*},\beta^{*}\right)$ *is the global minimizer of*


(9) $$\begin{array}{l} \underset{f}{\inf}\int_{-\infty }^{\infty }f^{\prime \prime }{\left(x\right)}^{2}\text{d}x\text{ s.t. }y_{n}=f\left(x_{n}\right)\forall n\in \left[N\right], \end{array}$$

*As such*, ${\hat{f}}_{\infty }\left(x;\mu^{*},\beta^{*}\right)$ *is a natural cubic smoothing spline with* *N* *degrees of freedom* (Ahlberg et al., 1967).

Theorem 5 of Williams et al. (2019), is a generalization of Corollary 4 to the non-uniform case which replaces Equation (9) with


$$\underset{f}{\inf}\int_{-\infty }^{\infty }\frac{f^{\prime \prime }{\left(x\right)}^{2}}{p_{\beta }\left(x\right)}\text{d}x\text{ s.t. }y_{n}=f\left(x_{n}\right)\forall n\in \left[N\right],$$

where *p*_β_(β) is the initial density of breakpoints induced by the specific initialization used.^2^ Deriving this result using the BDSO framework allows us to see that the initialization has the impact of weighting the curvature of certain locations more than others.

#### 2.6.2. Kernel Regime Dynamics as PDE

Assume a flat (μ_*i*_(0) = 0 ∀*i* ∈ [*H*]) kernel regime initialization. Specializing our spline parameterization dynamics results (Theorem 6), we have


μ˙i(t)=-〈ϵ^i(t),x〉+〈ϵ^i(t),1〉βi          ≜r1,i(t)+rx,i(t)βi.

Note that the terms *r*_1, *i*_(*t*) and *r*_*x, i*_(*t*) only depend on the index *i* through the mask $1_{i}$; in other words, they are the same for all breakpoints with the same activation pattern. This pattern is entirely determined by the orientation *s*_*i*_ and the data interval (*x*_*n*_, *x*_*n*+1_) that β_*i*_ falls into.

Thus, the possible activation patterns can be indexed by data point index *n* and orientation *s*. Letting ς_*n, s*_ denote the set of breakpoints with the activation pattern corresponding to (*n, s*), we have


$${\dot{\mu }}_{i}\left(t\right)=r_{1,n,s}\left(t\right)+r_{x,n,s}\left(t\right)\beta_{i}\;i\in \varsigma_{n,s}$$

Let ${\text{r}}_{1,n,s}\left(t\right)$ and ${\text{r}}_{x,n,s}\left(t\right)$ be vectors containing |ς_*n, s*_| copies of *r*_1, *n, s*_(*t*) (resp. *r*_*x, n, s*_(*t*)), and let ${\text{r}}_{1}\left(t\right)$ and ${\text{r}}_{x}\left(t\right)$ be the concatenation of these over all *n* and *s*. This allows us to write


$$\dot{\mu }\left(t\right)={\text{r}}_{1}\left(t\right)+{\text{r}}_{x}\left(t\right)⊙\beta ,$$

where μ and β are the vectors of μ_*i*_ (resp. β_*i*_) values for all *i*. Then, μ(t) can be viewed as a function μ:[H]×{+,-}×ℝ→ℝ, which is isomorphic to the function μ:ℝ × {+, −} × ℝ → ℝ given by $\mu \left(x,s,t\right)≜\overset{H}{\underset{i=1}{\sum }}\mu_{i}\left(t\right)\delta \left(x-\beta_{i}\right)$, yielding the PDE


(10) $$\begin{array}{l} \dot{\mu }\left(x,s,t\right)=r_{1}\left(x,s,t\right)+r_{x}\left(x,s,t\right)x, \end{array}$$

where *r*_1_(*x, s, t*) and *r*_*x*_(*x, s, t*) are piecewise constant functions of *x* with discontinuities at datapoints. Because *r*_1_(*x, s, t*) is piecewise constant in *x* for all *t*, $\int_{0}^{t}r_{1}\left(x,s,\tau \right)\text{d}\tau$ will also be piecewise constant, and likewise for *r*_*x*_(*x, s, t*). Thus, μ(*x, s, t*) will be (discontinuous) piecewise linear in *x* for all *t*. Stepping back, we see that a finite-width ReLU network trained by gradient descent is a discretization (in both space and time) of this underlying continuous PDE.

Thus, $\hat{f}$”_∞_(*x*; *t*) = μ(*x*, +, *t*)+μ(*x*, −, *t*), and as μ(*x, s, t*) is (discontinuous) piecewise linear, this implies that ${\hat{f}}_{\infty }\left(x;t\right)$ will be continuous piecewise cubic in *x* for all *t*, consistent with Corollary 4.

#### 2.6.3. Adaptive Regime

Previous work (Arora et al., 2019a) has shown that the kernel regression regime is insufficient to explain the generalization achieved by modern DL. Instead, the non-convexity of the optimization (i.e., the adaptive regime, α → 0, called the “rich regime” in Woodworth et al., 2020) must be responsible. The spline parametrization clearly separates the two, with all adaptive regime effects due to breakpoint dynamics β_*i*_(*t*): as α → 0, breakpoints become more mobile, and the breakpoint dynamics described in section 2.5 becomes more and more dominant. As shown in section 2.3, high breakpoint mobility can lead to clusters of breakpoints forming at certain specific data points. For low enough α, this clustering of breakpoints yielding a fit that is more linear spline than cubic spline; intermediate values of α interpolates these two extremes, giving a fit that has higher curvature in some areas than others.

Intriguingly, this suggests an explanation for why the kernel regime is insufficient: the adaptive regime enables breakpoint mobility, allowing the NN to adjust the initial breakpoint distribution (and thus, basis of activation patterns). This suggests that data-dependent breakpoint initializations may be quite useful (see Table 1 for a simple experiment in this vein).

**Table 1:** Test loss for standard vs. uniform breakpoint initialization, on sine and quadratic $\frac{x^{2}}{2}$.

**Init**	**Sine**	**Quadratic**
Standard	4.096 ± 2.25	0.1032 ± 0404
Uniform	2.280 ± 0.457	0.1118 ± 0.0248

Spline theory traditionally places a knot at each datapoint for smoothing splines (James et al., 2013). However, in circumstances where this is not feasible, either computationally or due to using a different spline, a variety of methods exist (MLE, at set quantiles of the regression variable, greedy algorithms, and more) (Park, 2001; Ruppert, 2002; Walker et al., 2005). More recent work has started to develop adaptive splines, where knots are placed to minimize roughness, length, or a similar metric, and knot locations are found via Monte Carlo methods or evolutionary algorithms (Miyata and Shen, 2005; Goepp et al., 2018). This adaptivity allows the spline to better model the function by moving knots to areas of higher curvature – remarkably similar to the behavior of a low-α NN.

In general, an individual breakpoint does not have a large impact on the overall function (*O*(1/*H*) on average); it is only through the cooperation of many breakpoints that large changes to the function are made. Another way of formulating this distinction is that the function (and hence the loss) depend on the breakpoints only through the empirical joint density ${\hat{p}}_{H}\left(\beta ,\mu ,s\right)$. Similarly, training dynamics depend on the θ_NN_ joint density ${\hat{p}}_{H}\left(w,v,b\right)$. This formulation is explored in Mei et al. (2018), where they derive a dynamics equation for the joint density (in θ_NN_). Adapting this work to explore the dynamics of the θ_BDSO_ joint density is ongoing work.

#### 2.6.4. Comparison With Concurrent Work

Independent of and concurrent with previous versions (Sahs et al., 2020a,b) of this work, Williams et al. (2019) has implicit regularization results in the kernel and adaptive regimes which parallel our results in this section rather closely. Despite the similarities, we take a significantly different approach. Comparing our results to those in Williams et al. (2019), the key differences are: (1) our BDSO parametrization has a clear geometric/functional interpretation whereas Williams *et al*.'s canonical parameters are opaque. (2) BDSO generalizes straightforwardly to high dimensions in a conceptually clean manner: oriented breakpoints become oriented breakplanes (see section 2.7); it is not clear what the multivariate analog of the canonical parameters would be. (3) Lemma 2 of Williams et al. (2019) and the surrounding text discuss the existence of what we call Attractor/Repulsor knot datapoints, but only gives an algebraic expression for their formation in terms of residuals and are unable to distinguish between the types, whereas we relate the persistence of such Attractors to the curvature of the ground truth function, particularly when breakpoints cross datapoints. (4) Our proof techniques are quite different from theirs, with different intuitions and intermediate results, and are also conceptually simpler. (5) Theorem 5 of Williams et al. (2019) is slightly more general form of our Corollary 4, extending to the case of non-uniform breakpoints. (6) Finally, we have many novel results regarding initialization, loss surface properties, Hessian and dynamics (everything outside of section 2.6). All in all, we believe our results are quite complementary to those of Williams et al., particularly as we extend our results to novel areas using our Hessian and loss surface analysis.

### 2.7. Extending to Multivariate Inputs

Throughout this work we chose to focus on the univariate case in order to build intuition and enable simpler theoretical results. However, in practice most networks have multivariate inputs; fortunately, our BDSO parametrization can be easily extended to multivariate inputs. For *D*-dimensional inputs, write


$$\hat{f}\left(\text{x};\theta_{\text{NN}}\right)=\overset{H}{\underset{i=1}{\sum }}v_{i}{\left(\langle {\text{w}}_{i},\text{x}\rangle +b_{i}\right)}_{+}+b_{0},$$

where the input weights are represented as *D*-dimensional vectors ${\text{w}}_{i}$. Using the reparametrization $\eta_{i}≜v_{i}{\left\|{\text{w}}_{i}\right\|}_{2}$, $\xi_{i}≜\frac{{\text{w}}_{i}}{{\left\|{\text{w}}_{i}\right\|}_{2}}$, $\gamma_{i}≜\frac{-b_{i}}{{\left\|{\text{w}}_{i}\right\|}_{2}}$, the representation becomes


$$\hat{f}\left(\text{x};\theta_{\text{BDSO}}\right)=\overset{H}{\underset{i=1}{\sum }}\eta_{i}{\left(\langle \xi_{i},\text{x}\rangle -\gamma_{i}\right)}_{+}+b_{0},$$

where η_*i*_ is a “delta-slope” parameter,^3^ and $\left(\xi_{i},\gamma_{i}\right)$ parametrize the orientation and signed distance from the origin of a *D* − 1-dimensional “oriented breakplane” (generalizing the 0-dimensional left-or-right oriented breakpoint represented by (*s*_*i*_, β_*i*_) in the 1-dimensional case). Generalizing our results to this parametrization is ongoing work.

## 3. Experiments

### 3.1. Suboptimality of Gradient Descent

**Figure 9:**
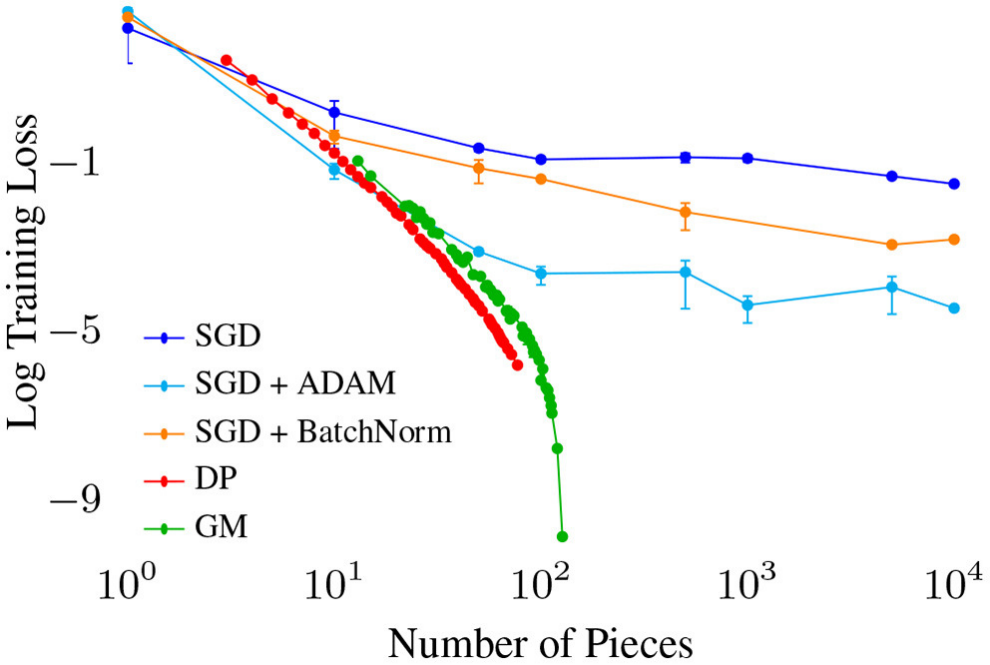
Training loss vs. number of pieces (∝ number of parameters) for various algorithms fitting a piece-wise linear (PWL) function to a quadratic. We observe a strict ordering of optimization quality with for the stochastic gradient descent based algorithms, with Adam (an optimizer with momentum) outperforming BatchNorm SGD outperforming Vanilla SGD.

Focusing on univariate networks allows us to directly compare against existing (near-) optimal algorithms for fitting Piecewise Linear (PWL) functions, including the Dynamic Programming algorithm (DP, Bai and Perron, 1998), and a very fast greedy approximation known as Greedy Merge (GM, Acharya et al., 2016) (Figure 9). Given a fixed budget of pieces (∝ to number of parameters e.g., network width), how well do these algorithms compare to SGD variants in fitting a quadratic (high curvature) function? DP and GM both quickly reduce training error to near 0 with order 100 pieces, with GM requiring slightly more pieces for similar performance. All the GD variants require far more pieces to reduce error below any target threshold, and may not even monotonically decrease with number of pieces. These results show how inefficient GD is w.r.t parameters, requiring orders of magnitude more for similar performance compared with PWL fitting algorithms.

### 3.2. Effect of Initial Breakpoint Distribution

We first ask whether the standard initializations will experience difficulty fitting functions that have significant curvature away from the origin (e.g., learning the energy function of a protein molecule). We train ReLU networks to fit a periodic function (sin(*x*)), which has high curvature both at and far from the origin. We find that the standard initializations do quite poorly away from the origin (Table 1, first row), consistent with our theory that breakpoints are essential for modeling curvature. Probing further, we observe empirically that breakpoints cannot migrate very far from their initial location, even if there are plenty of breakpoints overall, leading to highly suboptimal fits. In order to prove that it is indeed the breakpoint density that is causally responsible, we attempt to rescue the poor fitting by using a simple data-dependent initialization that samples breakpoints uniformly over the training data range [*x*_*min*_, *x*_*max*_], achieved by exploiting eq. (1). We train shallow ReLU networks on training data sampled from a sine and a quadratic function, two extremes on the spectrum of curvature. The data shows that uniform breakpoint density (Table 1, second row) rescues bad fits in cases with significant curvature far from the origin, with less effect on other cases, confirming the theory. We note that this could be a potentially useful data-dependent initialization strategy, one that can scale to high dimensions, but we leave this for future work.

### 3.3. Generalization: Implicit Regularization Emerges From Flat Init and Curvature-Based Parametrization

**Figure 10:**
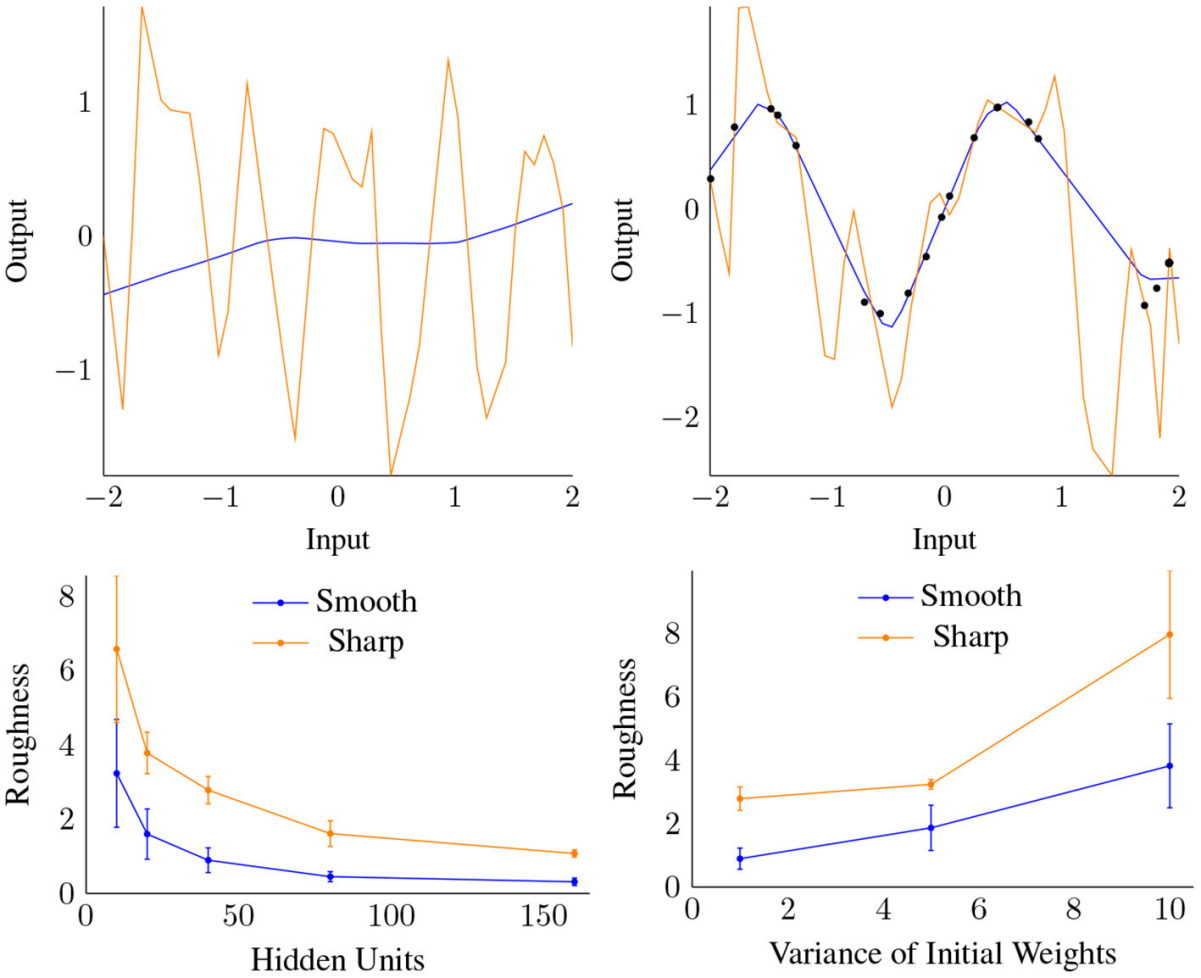
Top: “Spiky” (orange) and standard initialization (blue), compared before (left) and after (right) training. Note both cases reached similar, very low training set error. Bottom: Roughness vs. Width (left) and the variance of the initialization (right) for both data gap cases shown in Figure 11. Each datapoint is the result of averaging over 4 trials trained to convergence.

Our theory predicts that IR depends critically upon flatness of the initialization (Theorem 7 and corollary 4). Here, we test this theory for the case of shallow and deep univariate ReLU nets. We compare training with the standard flat initialization to a “spiky” initialization, and find that both fit the training data near perfectly, but that the “spiky” initialization has much worse generalization error (Figure 10 Top and Table 3 in Appendix).

It appears that generalization performance is not automatically guaranteed by GD, but is instead due in part to the flat initializations which are then *preserved* by GD. Our theoretical explanation is simple: integrating the dynamics in Equation (7) yields μ_*i*_(*t*) = μ_*i*_(0)+⋯  and so the initialization's impact remains.

### 3.4. Impact of Width and Init Variance

**Figure 11:**
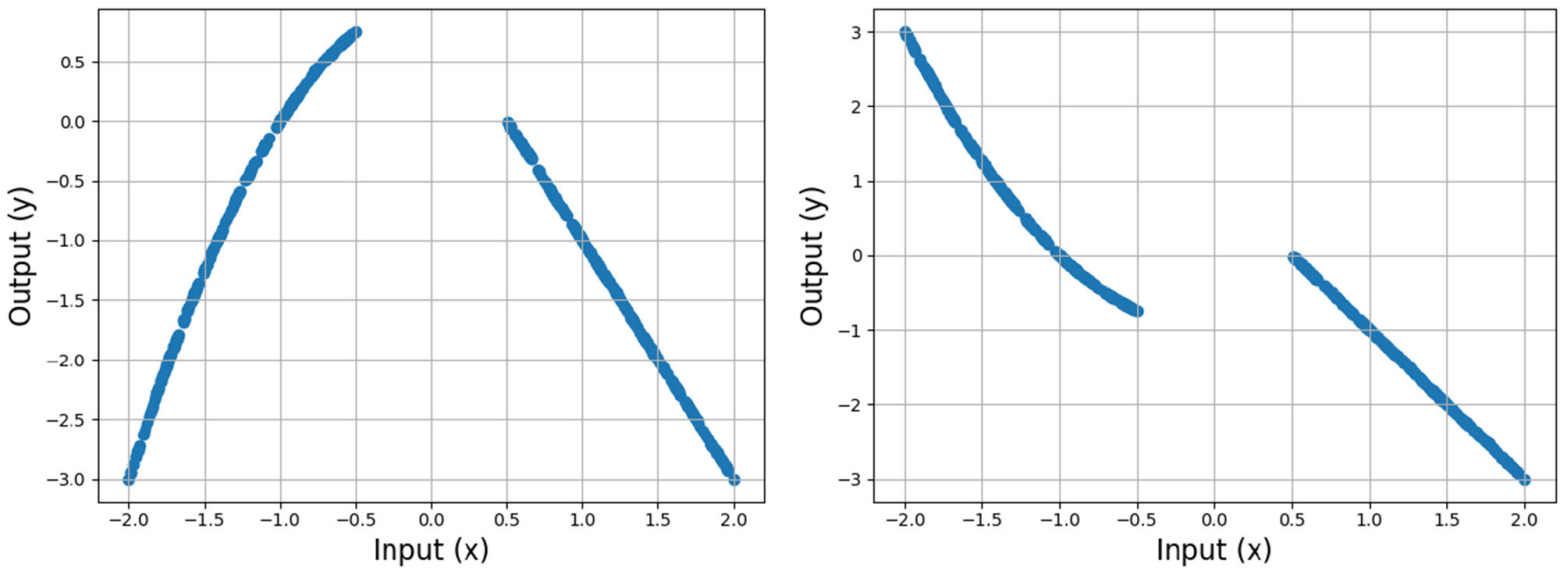
Training data sampled from two ground truth functions, one smoothly (left) and the other sharply (right) discontinuous, each with a data gap at [−0.5, 0.5].

Next, we examine how smoothness (roughness) depends on the width *H*, focusing on settings with large gaps in the training data. We use two discontinuous target functions (shown in Figure 11), leading to a gap in the data, and test how increasing *H* (with α unchanged) affects the smoothness of $\hat{f}$. We test this on a “smooth” data gap that is easily fit, as well as a “sharp” gap, where the fit will require a sharper turn. We trained shallow ReLU networks with varying width *H* and initial weight variance σ_*w*_ until convergence, and measured the total roughness of resulting CPWL approximation in the data gaps.

Figure 10 Bottom shows that roughness in the data gaps decreases with width and increases with initial weight variance, confirming our theory. A higher weight variance, and thus rougher initialization, acts in a similar fashion to the “spiky” initialization, and leads to increased roughness at convergence. In contrast, higher width distributes the curvature “work” over more units, leading to lower overall roughness.

### 3.5. Impact of Init Scale α

Finally, we explore how changing α impacts IR. Empirically, as α increases from 0 to ∞ we see three qualitative regimes: (i) an *under*fitting linear spline, (ii) an interpolating linear spline, and (iii) a roughness-minimizing natural cubic spline. This is quantified in Table 2, where we compare the NN fit to a linear interpolation and a natural cubic spline fit, for varying α. We first test in the special case that the initial function approximation is perfectly flat; we find excellent agreement with the linear interpolation and cubic spline fits for α = 3, 100 (Uniform initialization) and α = 10, 100 (He initialization). The impact of α on IR can be more easily visualized in our Supplementary Videos. In order to gauge the impact of the initialization breakpoint distribution, we also test with a standard He initialization (Cauchy distributed breakpoints, Corollary 1). In this case, we find that generalization error is uniformly higher for all α. More strikingly, the α regime (ii) above disappears, as a result of breakpoints being more concentrated around the origin and the initialization roughness being significantly nonzero. This supports the idea that the initial parameter settings, in particular the breakpoint distribution, has a critical impact on the final fit and its IR.

**Table 2:** Comparison of neural network trained to near 0 training loss on random data against linear interpolation and natural cubic splines for varying α, with uniform initialization (top) and standard He (bottom).

**α**	**MAE vs. Linear**	**RMSE vs. Linear**	**MAE vs. Cubic**	**RMSE vs. Cubic**
0.1	0.251 ± 0.077	0.370 ± 0.11	0.326 ± 0.12	0.442 ± 0.16
1	0.137 ± 0.060	0.228 ± 0.074	0.199 ± 0.084	0.282 ± 0.12
3	0.0296 ± 0.0083	0.0749 ± 0.018	0.117 ± 0.034	0.158 ± 0.048
10	0.122 ± 0.027	0.157 ± 0.029	0.0341 ± 0.012	0.0481 ± 0.019
100	0.159 ± 0.042	0.210 ± 0.055	0.0299 ± 0.011	0.0501 ± 0.024
0.1	0.202 ± 0.079	0.320 ± 0.13	0.293 ± 0.11	0.418 ± 0.16
1	0.134 ± 0.064	0.233 ± 0.11	0.211 ± 0.10	0.308 ± 0.15
3	0.132 ± 0.065	0.239 ± 0.12	0.209 ± 0.089	0.329 ± 0.15
10	0.115 ± 0.046	0.163 ± 0.061	0.0884 ± 0.052	0.149 ± 0.11
100	0.161 ± 0.048	0.212 ± 0.055	0.0556 ± 0.015	0.0828 ± 0.021

*Mean ± s.d. over 5 random seeds*.

Taken together, our experiments strongly support that a smooth, flat initialization and overparametrization are both responsible for the phenomenon and strength of IR, while the initialization weight scale α critically determines the type of IR.

## 4. Discussion

We show that removing the α-scaling symmetry and examining neural networks in spline space enabled us to glean new theoretical and practical insights. The spline view highlights the importance of initialization: a smooth initial approximation is required for a smooth final solution. Fortunately, existing initializations used in deep learning practice approximate this property. In spline space, we also achieve a surprisingly simple and transparent view of the loss surface, its critical points, its Hessian, and the phenomenon of overparametrization. It clarifies how increasing width relative to data size leads with high probability to lonely data partitions, which in turn are more likely to reach global minima. The spline view also allows us to explain the phenomenon of implicit regularization, and how it arises due to overparametrization and the initialization scale α.

### 4.1. Related Work

Our approach is related to previous work (Frankle and Carbin, 2018; Arora et al., 2019b; Savarese et al., 2019) in that we wish to characterize parameterization and generalization. We differ from these other works by focusing on small width networks, rather than massively overparametrized or infinite width networks, and by using a spline parameterization to study properties such as smoothness of the approximated function. Previous work (Advani and Saxe, 2017) has hinted at the importance of low norm initializations, but the spline perspective allows us to prove implicit regularization properties in shallow networks. Finally, Williams et al. (2019) is closely related and is discussed in detail at the end of section 2.6.

### 4.2. Explanation for Correlation Between Flatness of Minima and Generalization Error

A key unexplained empirical observation has been that flatter local minima tend to generalize better (Li et al., 2018; Wei and Schwab, 2019). Our results above provide an explanation: as overparametrization O=H/N increases, the flatness of the local minima (as measured by the number of zero eigenvalues) increases (Corollary 3) and the smoothness of the implicitly regularized function (as measured by inverse roughness $\rho \left({\hat{f}}_{H}\right)\geq \rho \left({\hat{f}}_{\infty }\right)$) also increases. As previously shown (Wu et al., 2017), flatter and simpler local minima imply better generalization. Our work provides a parsimonious explanation for this: as we increase overparametrization, partitions become increasingly lonely, allowing for an increased redundancy in number of ways to exactly fit the training data (thus increasing the number of zero eigenvalues), while the inductive bias of gradient descent spreads the “work” (e.g., curvature changes due to delta-slopes) among many units, ensuring that each unit has a lesser effect and making the loss surface increasingly flat.

### 4.3. Future Work

Looking forward, there are still many questions to answer from the spline perspective: How does depth affect the expressive power, learnability, and IR? What kinds of regularization are induced in the adaptive regime and how do modern deep nets take advantage of them? How can data-dependent initializations of the breakpoints help rescue/improve the performance of GD? Can we design new global learning algorithms inspired based on breakpoint (re)allocation? We believe the BDSO perspective can help answer these questions.

## Data Availability Statement

The original contributions presented in the study are included in the article/Supplementary Material, further inquiries can be directed to the corresponding author/s.

## Author Contributions

JS led effort on theory section, with help from RP and AP. RP led effort on experiments, with help from all other authors. JS and RP were responsible for manuscript. All authors contributed to the article and approved the submitted version.

## Funding

RP and AP were supported by Intelligence Advanced Research Projects Activity (IARPA) *via* Department of Interior/Interior Business Center (DoI/IBC) contract number D16PC00003. JS and AP were supported by NIH grant no. 1UF1NS111692-01. RP and AP were supported by funding from NSF NeuroNex grant no. 1707400.

## Conflict of Interest

The authors declare that the research was conducted in the absence of any commercial or financial relationships that could be construed as a potential conflict of interest.

## Publisher's Note

All claims expressed in this article are solely those of the authors and do not necessarily represent those of their affiliated organizations, or those of the publisher, the editors and the reviewers. Any product that may be evaluated in this article, or claim that may be made by its manufacturer, is not guaranteed or endorsed by the publisher.

## Footnotes

^1^Although a “breakpoints near curvature” fit will be a good fit, that does not mean that Gradient Descent from a random initialization can find such a fit; see section 2.3.

^2^In the original formulation (Williams et al., 2019), the *p*_β_(*x*) term is defined much more opaquely as ν(*x*)≜∫|*w*|*p*(*b* = *ax*|*w*)d*p*(*w*).

^3^For *D* = 1, we have η_*i*_ = *v*_*i*_|*w*_*i*_|, differing from the delta-slope μ_*i*_ = *v*_*i*_*w*_*i*_ used in this paper by the sign of *w*_*i*_.
