# Supplementary material for: Shallow Univariate ReLU Networks as Splines: Initialization, Loss Surface, Hessian, and Gradient Flow Dynamics
Source: Front Artif Intell. 2022 May 11;5:889981. doi: 10.3389/frai.2022.889981 (PMC9131019; doi:10.3389/frai.2022.889981)
Supplement: Supplementary file 1 [file Data_Sheet_1.pdf]

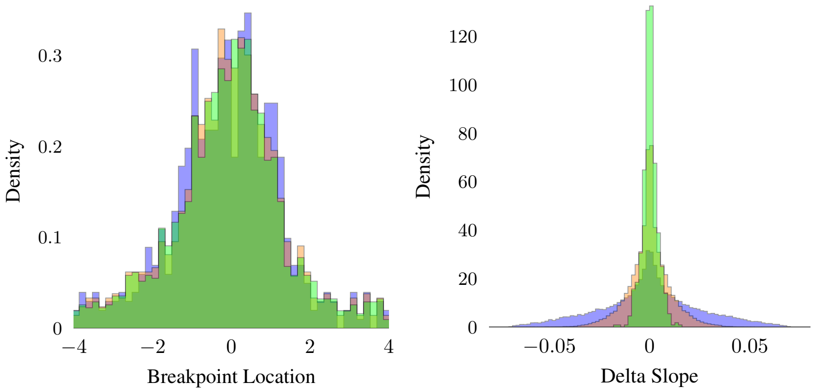

Figure 12. Left: Breakpoint distribution for a He initialization across a 3 layer network. Right: Delta-slope distribution for a He initialization across a 3 layer network.

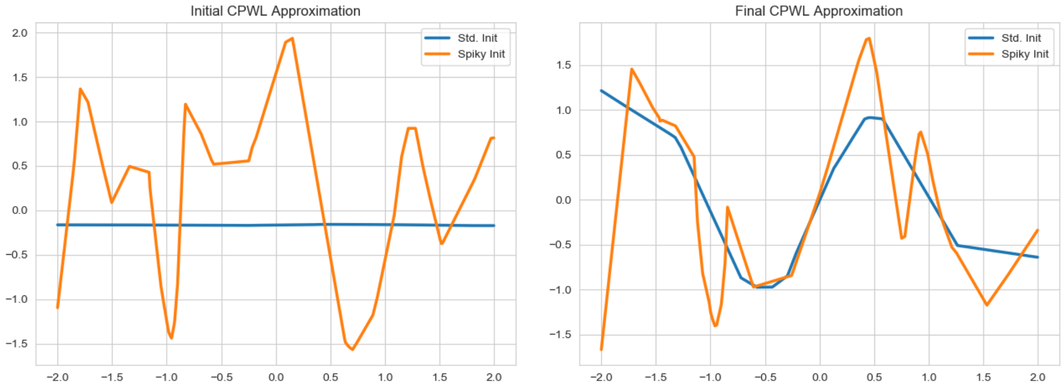

Figure 13. 'Spiky' (orange) and standard initialization (blue), compared before training (left) and post-training (right) using a deep network

5 APPENDIX

1 EXPERIMENTAL DETAILS

Breakpoint and delta-slope distributions at initialization. We first test our initialization theory against real networks. We initialize fully-connected ReLU networks of varying depths, according to the popular He initializations He et al. [2015]. Figure 12 shows experimentally measured densities of breakpoints and delta-slopes. Our theory matches the experiments well. The main points to note are that: (i) breakpoints are indeed more highly concentrated around the origin, and that (ii) as depth increases, delta-slopes have lower variance and thus lead to even flatter initial functions.

A.1 Uniform Initialization

Trained on a shallow, 21 unit FC ReLU network. Trained on function over the interval [-2,2]. Learning rate = 5e-5, trained via GD over 10000 epochs. Compared against pytorch default of He initialization. Training data sampled uniformly every .01 of the target interval. Each experiment was run 5 times, with results reported as mean $\pm$ standard deviation. Breakpoints y values were taken from the original standard initialization for the uniform initialization plus a small random noise term $\sim \mathcal{N}(0, .01)$, making initial condition within the target interval nearly identical.

Function	Shallow	Spiky Shallow	Deep	Spiky Deep
Sine	42.95 ± 6.406	157.5 ± 60.27	31.48 ± 7.078	122.0 ± 128.2
Arctan	0.013 ± 0.077	2.499 ± 1.257	0.980 ± 0.936	32.570 ± 26.100
Sawtooth	156.9 ± 12.45	150.1 ± 61.48	148.1 ± 8.755	198.0 ± 170.9
Cubic	3.608 ± 1.683	136.7 ± 124.1	56.77 ± 98.91	191.6 ± 114.1
Quadratic	3.559 ± 4.553	150.6 ± 49.00	1.741 ± 1.296	46.02 ± 19.42
Exp	$.6509 \pm .5928$	181.1 ± 75.36	1.339 ± 1.292	54.50 ± 37.77

Table 3. Comparison of testing loss of various network shallow and deep networks with a standard vs 'spiky' initialization

A.2 Spiky Initialization

For a shallow ReLU network, we can test a 'spiky' initialization by exactly solving for network parameters to generate a given arbitrary CPWL function. This network initialization is then compared against a standard initialization, and trained against a smooth function with a small number of training datapoints. The results of these experiments are shown in Table 3 and Figs. 10 and 13. Note that in a 1D input space we need a small number of training datapoints to create a situation similar to that of the sparsity caused by high dimensional input, and to allow for testing generalization between datapoints.

For a deep ReLU network, it is more difficult to exactly solve for a 'spiky' initialization. Instead, we train a network to approximate an arbitrary CPWL function, and call those trained network parameters the 'spiky' initialization. Once again, the 'spiky' initialization has near identical training performance, hitting all datapoints, but has noticeably worse generalization performance.

Detailed Experimental Procedure. Shallow version trained on a 21 unit FC ReLU Network. Deep version trained on a deep, 5-layer network with 4 hidden layers of width 8. In both cases, the 'spiky' initialization was a 20 - breakpoint CPWL function, with $y_n \sim \text{Uniform}([-2, 2])$. In the deep case, the spiky model was initialized with the same weights as the non-spiky model, and then pre-trained for 10,000 epochs to fit the CPWL. After that, gradient descent training proceeded on both models for 20,000 epochs, with all training having learning rate 1e-4. Training data was 20 random points in the range [-2,2], while the testing data (used to measure generalization) was spaced uniformly at every $\Delta x = .01$ of the target interval of the target function.

In the shallow case, there was no pre-training, as the 'spiky' model was directly set to be equal to the CPWL. In the shallow model, training occurred for 20,000 epochs. All experiment were run over 5 trials, and values in table are reported as mean $\pm$ standard deviation. Base shallow learning rate was 1e-4 using gradient descent method, with learning rate divided by 5 for the spiky case due to the initialization method generating larger weights. Despite differing learning rates, both models had similar training loss curves and similar final training loss values, e.g. for sine, final training loss was .94 for spiky and 1.02 for standard. Functions used were $\sin(x), \arctan(x)$, a sawtooth function from [-2,2] with minimum value of -1 at the endpoints, and 4 peaks of maximum value 1, cubic $\frac{x^3}{4} + \frac{x^2}{2} - \frac{x}{2}$, quadratic $\frac{x^2}{2}$, and $\exp(.5x)$ Note GD was chosen due to the strong theoretical focus of this paper - similar results were obtained using ADAM optimizer, in which case no differing learning rates were necessary.

A.3 Explaining the Need for Overparametrization

One possible explanation for why overparametrization is helpful is that it makes lonely partitions more likely to occur at initialization and remain lonely at subsequent times, resulting in a lonely final partition which can be trivially fit with 0 training loss. Figure 14 confirms the hypothesis above, for both our custom uniform and standard He initializations. If gradient descent training on neural networks must be

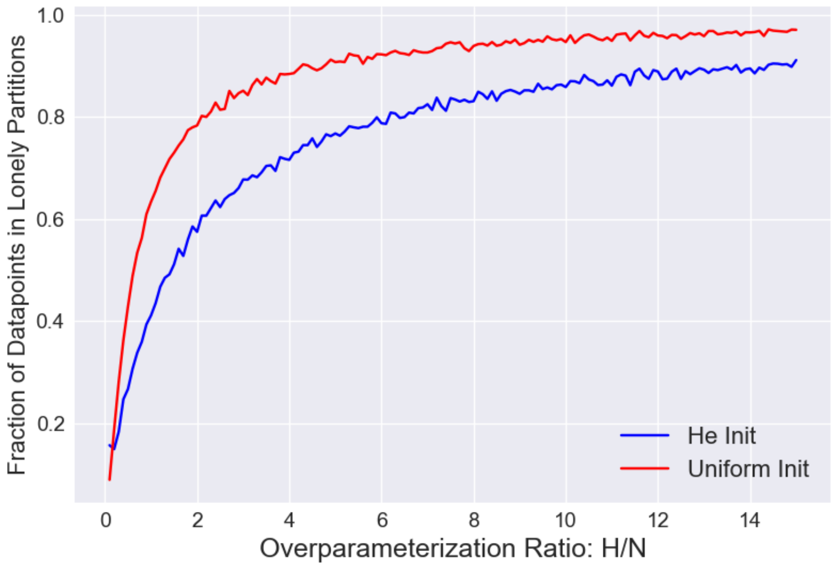

Figure 14. Percentage of datapoints which are in a lonely partition as a function of overparametrization ratio $\frac{H}{N}$ for both a standard (He) and uniform initializaion. Massive overparametrization leads w.h.p. to lonely partitions.

overparametrized to succeed, it implies that GD is highly inefficient with its parameters, leading us to ask the question: under what conditions will GD be successful?

Finally, we compare a standard He initialization vs our uniform initialization on a very small task. The He initialization leads to lonely partitions, preventing it from achieving training loss near 0 for $\alpha = 100$, while the uniform initialization with all lonely partitions is able to do so. These results can be seen in our Supplemental Videos.

A.4 Comparing Neural Network to Linear or Cubic Splines

In this section, we used a non-standard initialization in order to better compare with our theory. In particular, we set w to randomly be plus or minus 1 with equal probability, b to be a uniform partition of our x-range multiplied by the sign of w s.t. the breakpoints uniformly tile our x-range. v was multiplied by 0, leading to a perfectly flat initialization - note that as this is a shallow network, gradient can still flow through this after the first gradient descent update. Networks had hidden size $H = 1000$, and $N = 21$ datapoints were chosen with x values uniformly spaced from $[-2, 2]$, with an additional x datapoint at 3 and -3. y values for all datapoints were randomly chosen between $[-2, 2]$ independently. Prior to training, we constructed a linear interpolation of the data, as well as a natural cubic smoothing spline using the R method `smooth.spline`. α modification took place once before training began. Training was done using Adam, with a learning rate of 3e-5 for $\alpha \leq 1$, otherwise learning rate was divided by 5 to ensure that the networks had converged after 50,000 training epochs. The final neural network fit was compared against the spline fits over a fine grid with a Δx of .01 from -2 to 2. We considered the extra datapoints at $\pm$ 3 to act as 'pins' providing boundary conditions - without these, the neural network fits would still interpolate all interior datapoints, but potentially with different slopes through the first and last datapoints, leading to a sub-optimal fit.

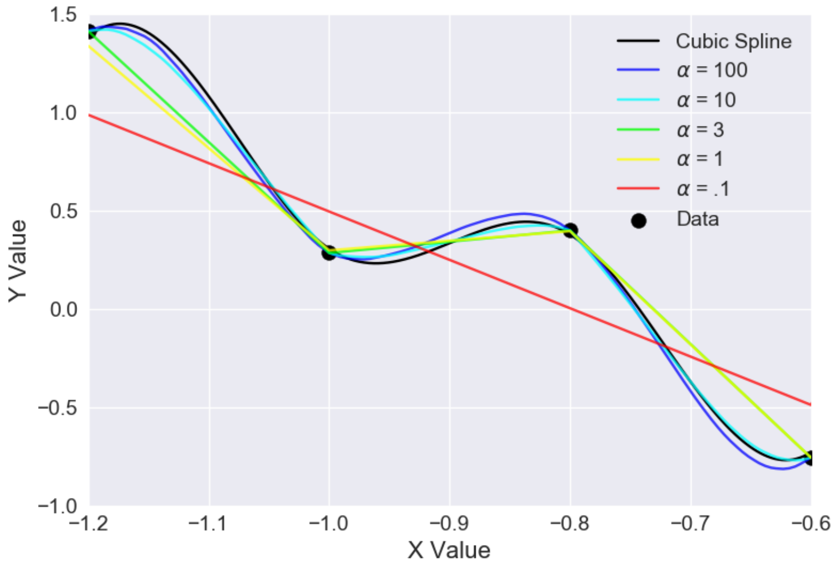

Figure 15. Effect of α, using same experimental protocol as Table 2, zoomed in on a small area of a single seed.

A.5 Supplemental Videos

We generate supplemental videos, using the settings in the above section at a fixed seed. We show both the general lower learning rate, and a faster learning rate version when necessary to show certain dynamics (i.e. for lower α). These videos are available at `http://shorturl.at/tFWZ2`.

A.6 Varying α

We look at a specific zoomed in portion of the trials above on a fixed seed, training to convergence, and show the results in Figure 15. This gives an intuitive picture of what varying α does, with too low giving an underfit, moderate giving a linear interpolation, and higher approaching the natural cubic spline.

2 PROOFS OF THEORETICAL RESULTS

B.1 Reparametrization from ReLU Network to Piecewise Linear Function

PROOF OF EQUATION (1):.

$$
\begin{aligned}
\hat{f}_{\theta,H}(x) &= \sum_{i=1}^{H} v_i \phi(w_i x + b_i) \\
&= \sum_{i=1}^{H} v_i (w_i x + b_i) [\![w_i x + b_i > 0]\!] \\
&= \sum_{i=1}^{H} v_i w_i (x - \beta_i) \begin{cases} [\![x > \beta_i]\!] & w_i > 0 \\ [\![x < \beta_i]\!] & w_i < 0 \end{cases} \quad \text{where } \beta_i \triangleq -\frac{b_i}{w_i} \\
&= \sum_{i=1}^{H} \mu_i (x - \beta_i) \begin{cases} [\![x > \beta_i]\!] & w_i > 0 \\ [\![x < \beta_i]\!] & w_i < 0 \end{cases} \quad \text{where } \mu_i \triangleq v_i w_i
\end{aligned}
$$

This gives us Equation (1), as desired.

B.2 Random Initialization in Function Space

Lemma 3. *Suppose (b_i, w_i, v_i) are initialized independently with densities $f_B(b_i)$, $f_W(w_i)$, and $f_V(v_i)$. Then, the density of (β_i, μ_i) is given by*

$$f_{\beta,\mu}(\beta_i, \mu_i) = \int_{-\infty}^{\infty} f_B(\beta_i u) f_W(u) f_V(\frac{\mu_i}{u})\, \mathrm{d}u\,.$$

PROOF. Suppose (b_i, w_i, v_i) are initialized i.i.d. from a distribution with density $f_{B,W,V}(b_i, w_i, v_i)$. Then, we can derive the density of (β_i, μ_i) by considering the invertible continuous transformation given by $(\beta_i, \mu_i, u) = g(b_i, w_i, v_i) = (b_i/w_i, v_i|w_i|, w_i)$, where $g^{-1}(\beta_i, \mu_i, u) = (\beta_i u, u, \mu_i/|u|)$. The density of (β_i, μ_i, u) is given by $f_{B,M,V}(\beta_i u, u, \mu_i/|u|)|J|$, where J is the Jacobian determinant of g^{-1}. Then, we have $J = -\operatorname{sgn} w_i$ and $|J| = 1$. The density of (β_i, μ_i) is then derived by integrating out the dummy variable u: $f_{\beta,\mu}(\beta_i, \mu_i) = \int_{-\infty}^{\infty} f_{B,W,V}(\beta_i u, u, \frac{\mu_i}{u})\, \mathrm{d}u$. If (b_i, w_i, v_i) are independent, this expands to $\int_{-\infty}^{\infty} f_B(\beta_i u) f_W(u) f_V(\frac{\mu_i}{u})\, \mathrm{d}u$.

B.3 Gaussian Initialization in Function

Theorem 1(a). *Consider a fully connected ReLU neural net with scalar input and output, and a single hidden layer of width H. Let the weights and biases be initialized randomly according to a zero-mean Gaussian or Uniform distribution. Then, under an independent Gaussian initialization,*

$$p_{\beta,\mu}(\beta_i, \mu_i) = \frac{1}{2\pi\sigma_v\sqrt{\sigma_b^2 + \sigma_w^2\beta_i^2}} \exp\left[-\frac{|\mu_i|\sqrt{\sigma_b^2 + \sigma_w^2\beta_i^2}}{\sigma_b\sigma_v\sigma_w}\right]$$

PROOF. Starting with Lemma 3,

$$\begin{aligned} f_{\beta,\mu}(\beta, \mu) &= \int_{-\infty}^{\infty} f_B(\beta_i u) f_W(u) f_V(\frac{\mu_i}{u})\, \mathrm{d}u \\ &= \int_{-\infty}^{\infty} \frac{1}{\sqrt{2\pi\sigma_b^2}} e^{-\frac{(\beta u)^2}{2\sigma_b^2}} \frac{1}{\sqrt{2\pi\sigma_w^2}} e^{-\frac{u^2}{2\sigma_w^2}} \frac{1}{\sqrt{2\pi\sigma_v^2}} e^{-\frac{(\mu/u)^2}{2\sigma_v^2}}\, du \\ \text{(Sympy)} &= \begin{cases} \dfrac{\exp\left[-\frac{\mu\sqrt{\sigma_b^2+\sigma_w^2(\beta)^2}}{\sigma_b\sigma_v\sigma_w}\right]}{2\pi\sigma_v\sqrt{\sigma_b^2+\sigma_w^2(\beta)^2}} & \mu > 0 \\ \text{unknown} & \text{otherwise} \end{cases} \end{aligned}$$

but the integrand is even in μ, giving

$$= \frac{\exp\left[-\frac{|\mu|\sqrt{\sigma_b^2+\sigma_w^2(\beta)^2}}{\sigma_b\sigma_v\sigma_w}\right]}{2\pi\sigma_v\sqrt{\sigma_b^2 + \sigma_w^2(\beta)^2}}$$

Corollary 1(a). *Consider the same setting as Theorem 1. In the case of an independent Gaussian initialization,*

$$p_\beta(\beta_i) = \textit{Cauchy}\left(\beta_i; 0, \frac{\sigma_b}{\sigma_w}\right) = \frac{\sigma_b\sigma_w}{\pi\left(\sigma_w^2\beta_i^2 + \sigma_b^2\right)}$$

$$p_\mu(\mu_i) = \frac{1}{2\pi\sigma_v\sigma_w} G_{0,2}^{2,0}\left(\frac{\mu_i^2}{4\sigma_v^2\sigma_w^2}\middle| 0, 0\right) = \frac{1}{\pi\sigma_v\sigma_w} K_0\left(\frac{|\mu_i|}{\sigma_v\sigma_w}\right)$$

$$p_{\mu|\beta}(\mu_i|\beta_i) = \textit{Laplace}\left(\mu_i; 0, \frac{\sigma_b\sigma_v\sigma_w}{\sqrt{\sigma_b^2 + \sigma_w^2\beta_i^2}}\right) = \frac{\sqrt{\sigma_b^2 + \sigma_w^2\beta_i^2}}{2\sigma_b\sigma_v\sigma_w} \exp\left[-\frac{|\mu_i|\sqrt{\sigma_b^2 + \sigma_w^2\beta_i^2}}{\sigma_b\sigma_v\sigma_w}\right],$$

where $G_{pq}^{nm}(\cdot|\cdot)$ is the Meijer G-function and $K_\nu(\cdot)$ is the modified Bessel function of the second kind.

PROOF. Marginalizing out μ from the joint density in Sympy returns the desired $f_\beta(\beta)$ from above. Sympy cannot compute the other marginal, so we verify it by hand:

$$f_\mu(\mu) = \int_{-\infty}^{\infty} \frac{\exp\left[-\frac{|\mu|\sqrt{\sigma_b^2+\sigma_w^2\beta^2}}{\sigma_b\sigma_v\sigma_w}\right]}{2\pi\sigma_v\sqrt{\sigma_b^2 + \sigma_w^2\beta^2}} dx = \frac{1}{2\pi\sigma_v}\int_{-\infty}^{\infty} \frac{\exp\left[-\frac{|\mu|\sqrt{\sigma_b^2+\sigma_w^2\beta^2}}{\sigma_b\sigma_v\sigma_w}\right]}{\sqrt{\sigma_b^2 + \sigma_w^2\beta^2}} dx$$

$$\left(\phi(\beta) = \frac{\beta}{\sigma_w}\right) \qquad = \frac{1}{2\pi\sigma_v \underbrace{\sigma_w}_{\phi'(\beta)}}\int_{-\infty}^{\infty} \frac{\exp\left[\frac{-|\mu|\sqrt{\sigma_b^2+\beta^2}}{\sigma_b\sigma_v\sigma_w}\right]}{\sqrt{\sigma_b^2 + \beta^2}} dx$$

from Gradshteyn and Ryzhik [2015], Eq. 3.462.20, we have

$$K_0(ab) = \int_0^\infty \frac{\exp\left(-a\sqrt{\beta^2+b^2}\right)}{\sqrt{\beta^2+b^2}} \mathrm{d}\beta \qquad [\mathrm{Re}\, a > 0, \mathrm{Re}\, b > 0]$$

$$\text{(integrand is even in } \beta) = \frac{1}{2}\int_{-\infty}^\infty \frac{\exp\left(-a\sqrt{\beta^2+b^2}\right)}{\sqrt{\beta^2+b^2}} \mathrm{d}\beta \qquad [\mathrm{Re}\, a > 0, \mathrm{Re}\, b > 0]$$

applying this with $a = \frac{|\mu|}{\sigma_b\sigma_v\sigma_w}$ and $b = \sigma_b$,

$$\frac{1}{2\pi\sigma_v\sigma_w}\int_{-\infty}^{\infty} \frac{\exp\left[\frac{-|\mu|\sqrt{\sigma_b^2+\beta^2}}{\sigma_b\sigma_v\sigma_w}\right]}{\sqrt{\sigma_b^2 + \beta^2}} \mathrm{d}\beta = \frac{1}{\pi\sigma_v\sigma_w} K_0\left(\frac{|\mu|}{\sigma_v\sigma_w}\right)$$

as desired. We can then use these densities to derive the conditional:

$$f_\mu(\mu|\beta) = \frac{\sqrt{\sigma_b^2 + \sigma_w^2(\beta)^2}\exp\left[-\frac{|\mu|\sqrt{\sigma_b^2+\sigma_w^2(\beta)^2}}{\sigma_b\sigma_v\sigma_w}\right]}{2\sigma_b\sigma_v\sigma_w}$$
$$= \text{Laplace}\left(\mu; 0, \frac{\sigma_b\sigma_v\sigma_w}{\sqrt{\sigma_b^2 + \sigma_w^2(\beta)^2}}\right).$$

B.4 Uniform Initialization in Function Space

Theorem 1(b). *Consider a fully connected ReLU neural net with scalar input and output, and a single hidden layer of width H. Let the weights and biases be initialized randomly according to a zero-mean Gaussian or Uniform distribution. Then, under an independent Uniform initialization,*

$$p_{\beta,\mu}(\beta_i, \mu_i) = \frac{[\![|\mu_i| \le \min\{\frac{a_b a_v}{|\beta_i|}, a_w, a_v\}]\!]}{4a_b a_w a_v}\left(\min\{\frac{a_b}{|\beta_i|}, a_w\} - \frac{|\mu_i|}{a_v}\right)$$

PROOF. Starting with Lemma 3,

$$f_{\beta,\mu}(\beta,\mu) = \int_{-a_w}^{a_w} f_B(\beta u) f_W(u) f_V(\mu/u)\,\mathrm{d}u$$
$$= \int_{-a_w}^{a_w} \frac{1}{2a_b}[\![-a_b \le \beta u \le a_b]\!]\frac{1}{2a_w}[\![-a_w \le u \le a_w]\!]\frac{1}{2a_v}[\![-a_v \le \mu/u \le a_v]\!]\,\mathrm{d}u$$
$$= \int_{-a_w}^{a_w} \frac{1}{2a_b}[\![-a_b/|\beta| \le u \le a_b/|\beta|]\!]\frac{1}{2a_w}[\![-a_w \le u \le a_w]\!]\frac{1}{2a_v}[\![u \le -|\mu|/a_v \vee u \ge |\mu|/a_v]\!]\,\mathrm{d}u$$
$$= \int_{-a_w}^{a_w} \frac{1}{8a_b a_w a_v}[\![-\min\{a_b/|\beta|, a_w\} \le u \le -|\mu|/a_v \vee |\mu|/a_v \le u \le \min\{a_b/|\beta|, a_w\}]\!]$$
$$\times[\![|\mu| \le a_b a_v/|\beta|]\!]\,\mathrm{d}u$$
$$= \frac{[\![|\mu| \le a_b a_v/|\beta|]\!]}{8a_b a_w a_v}\int_{-a_w}^{a_w}[\![-\min\{a_b/|\beta|, a_w\} \le u \le -|\mu|/a_v \vee |\mu|/a_v \le u \le \min\{a_b/|\beta|, a_w\}]\!]\,\mathrm{d}u$$
$$= \frac{[\![|\mu| \le a_b a_v/|\beta|]\!]}{4a_b a_w a_v}\int_0^{a_w}[\![|\mu|/a_v \le u \le \min\{a_b/|\beta|, a_w\}]\!]\,\mathrm{d}u$$
$$= \frac{[\![|\mu| \le a_b a_v/|\beta|]\!]}{4a_b a_w a_v}\left(\min\{a_b/|\beta|, a_w\} - |\mu|/a_v\right)[\![-a_w a_v \le \mu \le a_w a_v]\!]$$

as desired.

Corollary 1(b). *Consider the same setting as Theorem 1. In the case of an independent Uniform initialization,*

$$p_\beta(\beta_i) = \frac{1}{4a_b a_w}\left(\min\left\{\frac{a_b}{|\beta_i|}, a_w\right\}\right)^2$$
$$p_\mu(\mu_i) = \frac{[\![-a_w a_v \le \mu_i \le a_w a_v]\!]}{2a_w a_v}\log\frac{a_w a_v}{|\mu_i|}$$
$$p_{\mu|\beta}(\mu_i|\beta_i) = \mathit{Tri}(\mu_i; a_v \min\{a_b/|\beta_i|, a_w\}) = \frac{[\![|\mu_i| \le a_v \min\{a_b/|\beta_i|, a_w\}]\!]}{a_v \min\{a_b/|\beta_i|, a_w\}}\left(1 - \frac{|\mu_i|}{a_v \min\{a_b/|\beta_i|, a_w\}}\right),$$

where Tri$(\cdot; a)$ *is the symmetric triangular distribution with base* $[-a, a]$ *and mode* 0.

PROOF. Beginning with the marginal of β_i,

$$\begin{aligned}
f_\beta(\beta) &= \int f_{\beta,\mu}(\beta,\mu)\,\mathrm{d}\mu \\
&= \int_{-\infty}^{\infty} \frac{[\![|\mu| \le a_b a_v/|\beta|]\!]}{4a_b a_w a_v}\left(\min\{a_b/|\beta|, a_w\} - |\mu|/a_v\right)[\![-a_w a_v \le \mu \le a_w a_v]\!]\,\mathrm{d}\mu \\
&= \int_{-a_w a_v}^{a_w a_v} \frac{[\![|\mu| \le a_b a_v/|\beta|]\!]}{4a_b a_w a_v}\left(\min\{a_b/|\beta|, a_w\} - |\mu|/a_v\right)\mathrm{d}\mu \\
&= \frac{1}{2a_b a_w a_v}\int_0^{a_w a_v} [\![\mu \le a_b a_v/|\beta|]\!]\left(\min\{a_b/|\beta|, a_w\} - \mu/a_v\right)\mathrm{d}\mu \\
&= \frac{1}{2a_b a_w a_v}\left(\int_0^{a_w a_v} [\![\mu \le a_b a_v/|\beta|]\!]\min\{a_b/|\beta|, a_w\}\,\mathrm{d}\mu - \int_0^{a_w a_v}[\![\mu \le a_b a_v/|\beta|]\!]\mu/a_v\,\mathrm{d}\mu\right) \\
&= \frac{1}{2a_b a_w a_v}\left(\min\{a_b/|\beta|, a_w\}\int_0^{a_w a_v}[\![\mu \le a_b a_v/|\beta|]\!]\,\mathrm{d}\mu - \frac{1}{a_v}\int_0^{a_w a_v}[\![\mu \le a_b a_v/|\beta|]\!]\mu\,\mathrm{d}\mu\right) \\
&= \frac{1}{2a_b a_w a_v}\left(\min\{a_b/|\beta|, a_w\}\min\{a_w a_v, a_b a_v/|\beta|\} - \frac{1}{a_v}\int_0^{\min\{a_w a_v, a_b a_v/|\beta|\}}\mu\,\mathrm{d}\mu\right) \\
&= \frac{1}{2a_b a_w a_v}\left(\min\{a_b/|\beta|, a_w\}\min\{a_w a_v, a_b a_v/|\beta|\} - \frac{1}{2a_v}\left(\min\{a_w a_v, a_b a_v/|\beta|\}\right)^2\right) \\
&= \frac{1}{2a_b a_w a_v}\left(a_v\left(\min\{a_b/|\beta|, a_w\}\right)^2 - \frac{1}{2a_v}\left(a_v\min\{a_w, a_b/|\beta|\}\right)^2\right) \\
&= \frac{1}{2a_b a_w a_v}\left(a_v\left(\min\{a_b/|\beta|, a_w\}\right)^2 - \frac{a_v}{2}\left(\min\{a_w, a_b/|\beta|\}\right)^2\right) \\
&= \frac{1}{4a_b a_w}\left(\min\{a_b/|\beta|, a_w\}\right)^2
\end{aligned}$$

as desired. Then,

$$
\begin{aligned}
f_\mu(\mu) &= \int f_{\beta,\mu}(\beta,\mu)\,\mathrm{d}\beta \\
&= \int_{-\infty}^{\infty} \frac{[\![|\mu| \le a_b a_v/|\beta|]\!]}{4a_b a_w a_v} \left(\min\{a_b/|\beta|, a_w\} - |\mu|/a_v\right) [\![-a_w a_v \le \mu \le a_w a_v]\!]\,\mathrm{d}\beta \\
&= \frac{[\![-a_w a_v \le \mu \le a_w a_v]\!]}{4a_b a_w a_v} \int_{-\infty}^{\infty} [\![|\mu| \le a_b a_v/|\beta|]\!] \left(\min\{a_b/|\beta|, a_w\} - |\mu|/a_v\right) \mathrm{d}\beta \\
&= \frac{[\![-a_w a_v \le \mu \le a_w a_v]\!]}{4a_b a_w a_v} 2 \int_{0}^{\infty} [\![|\mu| \le a_b a_v/\beta]\!] \left(\min\{a_b/\beta, a_w\} - |\mu|/a_v\right) \mathrm{d}\beta \\
&= \frac{[\![-a_w a_v \le \mu \le a_w a_v]\!]}{4a_b a_w a_v} 2 \int_{0}^{\infty} [\![\beta \le a_b a_v/|\mu|]\!] \left(\min\{a_b/\beta, a_w\} - |\mu|/a_v\right) \mathrm{d}\beta \\
&= \frac{[\![-a_w a_v \le \mu \le a_w a_v]\!]}{4a_b a_w a_v} 2 \int_{0}^{a_b a_v/|\mu|} \min\{a_b/\beta, a_w\} - |\mu|/a_v\,\mathrm{d}\beta \\
&= \frac{[\![-a_w a_v \le \mu \le a_w a_v]\!]}{4a_b a_w a_v} 2 \left(\int_{0}^{\min\{a_b a_v/|\mu|, a_b/a_w\}} a_w - |\mu|/a_v\,\mathrm{d}\beta \right. \\
&\qquad\qquad \left. + \int_{\min\{a_b a_v/|\mu|, a_b/a_w\}}^{a_b a_v/|\mu|} a_b/\beta - |\mu|/a_v\,\mathrm{d}\beta \right) \\
&= \frac{[\![-a_w a_v \le \mu \le a_w a_v]\!]}{4a_b a_w a_v} 2 \left(\int_{0}^{a_b/a_w} a_w - |\mu|/a_v\,\mathrm{d}\beta + \int_{a_b/a_w}^{a_b a_v/|\mu|} a_b/\beta - |\mu|/a_v\,\mathrm{d}\beta \right) \\
&= \frac{[\![-a_w a_v \le \mu \le a_w a_v]\!]}{4a_b a_w a_v} 2 \left((a_b/a_w)(a_w - |\mu|/a_v) + \int_{a_b/a_w}^{a_b a_v/|\mu|} a_b/\beta\,\mathrm{d}\beta - \int_{a_b/a_w}^{a_b a_v/|\mu|} |\mu|/a_v\,\mathrm{d}\beta \right) \\
&= \frac{[\![-a_w a_v \le \mu \le a_w a_v]\!]}{4a_b a_w a_v} 2 \left[(a_b/a_w)(a_w - |\mu|/a_v) + \int_{a_b/a_w}^{a_b a_v/|\mu|} a_b/\beta\,\mathrm{d}\beta \right. \\
&\qquad\qquad \left. - (|\mu|/a_v)(a_b a_v/|\mu| - a_b/a_w) \right] \\
&= \frac{[\![-a_w a_v \le \mu \le a_w a_v]\!]}{4a_b a_w a_v} 2 \left((a_b/a_w)(a_w - |\mu|/a_v) + a_b \log \frac{a_b a_v/|\mu|}{a_b/a_w} - (|\mu|/a_v)(a_b a_v/|\mu| - a_b/a_w) \right) \\
&= \frac{[\![-a_w a_v \le \mu \le a_w a_v]\!]}{4a_b a_w a_v} 2a_b \log \frac{a_b a_v/|\mu|}{a_b/a_w} \\
&= \frac{[\![-a_w a_v \le \mu \le a_w a_v]\!]}{2a_w a_v} \log \frac{a_b a_v/|\mu|}{a_b/a_w} \\
&= \frac{[\![-a_w a_v \le \mu \le a_w a_v]\!]}{2a_w a_v} \log \frac{a_w a_v}{|\mu|}
\end{aligned}
$$

as desired. We can then use these densities to derive the conditional:

$$
\begin{aligned}
f_\mu(\mu_i|\beta_i) &= \frac{\frac{[\![|\mu_i|\le a_b a_v/|\beta_i|]\!]}{4a_b a_w a_v}\left(\min\{a_b/|\beta_i|, a_w\} - |\mu_i|/a_v\right)[\![-a_w a_v \le \mu_i \le a_w a_v]\!]}{\frac{1}{4a_b a_w}\left(\min\{a_b/|\beta_i|, a_w\}\right)^2} \\
&= \frac{\frac{[\![|\mu_i|\le a_b a_v/|\beta_i|]\!][\![-a_w a_v\le\mu_i\le a_w a_v]\!]}{a_v}\left(\min\{a_b/|\beta_i *|, a_w\} - |\mu_i|/a_v\right)}{\left(\min\{a_b/|\beta_i|, a_w\}\right)^2} \\
&= \frac{[\![|\mu_i| \le a_v \min\{a_b/|\beta_i|, a_w\}]\!]}{a_v \min\{a_b/|\beta_i|, a_w\}}\left(1 - \frac{|\mu_i|}{a_v \min\{a_b/|\beta_i|, a_w\}}\right).
\end{aligned}
$$

Remarks. Note that the marginal distribution on μ_i is the distribution of a product of two independent random variables, and the marginal distribution on β_i is the distribution of the ratio of two random variables. For the Gaussian case, the marginal distribution on μ_i is a symmetric distribution with variance $\sigma_v^2\sigma_w^2$ and excess Kurtosis of 6. For the Uniform case, the marginal distribution of β_i is a symmetric distribution with no finite higher moments. The marginal distribution of μ_i is a symmetric distribution with bounded support and variance $\frac{2a_w^3 a_v^3}{9}$ and excess Kurtosis of $\frac{81}{50a_w a_v} - 3$. The conditional distribution of μ_i given β_i is a symmetric distribution with bounded support and variance $\frac{(a_v \min\{a_b/|\beta_i|, a_w\})^2}{6}$ and excess Kurtosis of $-\frac{3}{5}$.

B.5 Roughness of Random Initialization

Theorem 2. *Consider the initial roughness ρ_0 under a Gaussian initialization. In the He initialization, we have that the tail probability is given by*

$$
\mathbb{P}[\rho_0 - \mathbb{E}[\rho_0] \ge \lambda] \le \frac{1}{1 + \frac{\lambda^2 H}{128}},
$$

where $\mathbb{E}[\rho_0] = 4$. In the Glorot initialization, we have that the tail probability is given by

$$
\mathbb{P}[\rho_0 - \mathbb{E}[\rho_0] \ge \lambda] \le \frac{1}{1 + \frac{\lambda^2 (H+1)^4}{128H}},
$$

where $\mathbb{E}[\rho_0] = \frac{4H}{(H+1)^2} = O\left(\frac{1}{H}\right)$.

PROOF. Using the moments of the delta-slope distribution computed in Theorem 1, Corollary 1, and above, we can compute:

$$
\begin{aligned}
\mathbb{E}[\rho_0] &= \sum_{i=1}^{H} \mathbb{E}[\mu_i^2] = \sum_{i=1}^{H} \mathrm{Var}[\mu_i] + \mathbb{E}[\mu_i]^2 = H\sigma_v^2\sigma_w^2 \\
\mathrm{Var}[\rho_0] &= \sum_{i=1}^{H} \mathrm{Var}[\mu_i^2] = H\,\mathrm{Var}[\mu_i^2] = H(\mathbb{E}[\mu_i^4] - \mathbb{E}[\mu_i^2]^2) \\
&= H(9(\sigma_v\sigma_w)^4 - \sigma_v^4\sigma_w^4) = 8H\sigma_v^4\sigma_w^4
\end{aligned}
$$

Applying the two initializations, we have

$$
\begin{aligned}
\mathbb{E}[\rho_0^{\text{He}}] &= H\frac{2}{H}\frac{2}{1} = 4 \\
\text{Var}[\rho_0^{\text{He}}] &= 8H\frac{4}{H^2}\frac{4}{1} = \frac{128}{H} \\
\mathbb{E}[\rho_0^{\text{Glorot}}] &= H\frac{2}{H+1}\frac{2}{1+H} = \frac{4H}{(H+1)^2} = O\left(\frac{1}{H}\right) \\
\text{Var}[\rho_0^{\text{Glorot}}] &= 8H\frac{4}{(H+1)^2}\frac{4}{(1+H)^2} = \frac{128H}{(H+1)^4} = O\left(\frac{1}{H^3}\right)
\end{aligned}
$$

By applying Cantelli's theorem, we get the stated tail probabilities.

B.6 Loss Surface in the Spline Parametrization

Theorem 3. *The loss function $\tilde{\ell}(\theta_{BDSO} = (\boldsymbol{\beta}, \boldsymbol{\mu}, \mathrm{s}))$ is a continuous piecewise quadratic spline. Furthermore, consider the evolution of the loss as we vary β_i along the x axis; this 1-dimensional slice $\tilde{\ell}(\beta_i; \boldsymbol{\beta}_{-i}, \boldsymbol{\mu}, \mathrm{s})$ is also a continuous piecewise quadratic spline in β_i with knots at datapoints $\{x_n\}_{n=1}^N$. Let $p_1(\beta_i)$ (resp. $p_2(\beta_i)$) be the quadratic function equal to $\tilde{\ell}(\beta_i; \boldsymbol{\beta}_{-i}, \boldsymbol{\mu}, \mathrm{s})$ for $\beta_i \in [x_{n-1}, x_n]$ (resp. $[x_n, x_{n+1}]$), which both have positive curvature, and let $m_j \triangleq \arg\min p_j(\beta_i)$. Then, with measure 1, the knots x_n fall into one of three types as shown in Figure 3:*

-(Type I, Passthrough) $m_1, m_2 < x_n$, or $x_n < m_1, m_2$,

-(Type II, Repeller) $m_1 < x_n < m_2$

-(Type III, Attractor) $m_2 < x_n < m_1$.

PROOF. First, consider the following function:

$$
g(\boldsymbol{\beta}, \boldsymbol{\mu}) = \sum_{n=1}^{N} \left(\sum_{i=1}^{H} \mu_i (x_n - \beta_i) - y_n \right)^2 .
$$

As a sum of squares of linear terms, this is clearly quadratic in $(\boldsymbol{\beta}, \boldsymbol{\mu})$. Then, note that the loss

$$
\tilde{\ell}(\boldsymbol{\beta}, \boldsymbol{\mu}, \mathrm{s}) = \sum_{n=1}^{N} \left(\sum_{i=1}^{H} \mu_i (x_n - \beta_i)_{s_i} - y_n \right)^2
$$

differs only in the addition of the $(\cdot)_{s_i}$ operation, which converts each linear term to a continuous piecewise linear term. Thus, $\tilde{\ell}(\cdot)$ is continuous piecewise quadratic in $(\boldsymbol{\beta}, \boldsymbol{\mu})$. Furthermore, holding $\boldsymbol{\mu}$ and s constant, we still have the composition of a quadratic and piecewise linear function. In particular, the piecewise linear terms have their knots at $\beta_i = x_n$, which are inherited by $\tilde{\ell}(\cdot)$.

Finally, because $\tilde{\ell}(\cdot)$ is of the form $\sum_{n=1}^N \epsilon_n^2$, it has non-negative curvature everywhere, so m_1 and m_2 are well-defined for each knot. Then, we can analyze the loss surface under the various orderings of m_1, m_2, and x_n. We may ignore the case that $m_j = x_n$ as this is an event of measure 0. It is straightforward to collect the remaining cases into the three types depicted in Figure 3.

Theorem 4. *Consider some arbitrary θ^*_{NN} such that there exists at least one neuron that is active on all data. Then, θ^*_{NN} is a critical point of $\tilde{\ell}(\cdot)$ if and only if for all partitions $\mathcal{X}_p$, the restriction $\hat{f}(\cdot;\theta^*_{NN})|_{\mathcal{X}_p}$ is an Ordinary Least Squares (OLS) fit of the training data contained in π_p.*

PROOF. Let θ^*_{NN} be as hypothesized. Then, θ^*_{NN} is a critical point of $\tilde{\ell}(\cdot)$ iff $\left.\frac{\partial \tilde{\ell}(\cdot)}{\partial \theta_{\text{NN}}}\right|_{\theta^*_{\text{NN}}} = \mathbf{0}$. Expanding, we have that the right hand sides of Equation (5) are all 0. Without loss of generality, assume there is no $i \in [H]$ such that $v_i = w_i = b_i = 0$. Simplifying under our assumptions, we get

$$
\begin{aligned}
\langle \hat{\boldsymbol{\epsilon}}, \mathbf{1} \rangle &= 0 \\
\langle \hat{\boldsymbol{\epsilon}}_i, \mathbf{1} \rangle &= 0 \quad \forall i \in [H] \\
\langle \hat{\boldsymbol{\epsilon}}_i, \mathbf{x} \rangle &= 0 \quad \forall i \in [H]
\end{aligned}
$$

The latter two lines can be written as

$$
\left(\underset{N\times H}{\overline{\boldsymbol{\epsilon}}} \odot \underset{N\times H}{\hat{\mathbf{A}}} \right)^{\top} \underset{N\times 2}{\tilde{\mathbf{X}}} = \underset{H\times 2}{\mathbf{0}},
$$

where $\overline{\boldsymbol{\epsilon}}$ is H copies of the N-dimensional vector $\hat{\boldsymbol{\epsilon}}$, $\hat{a}_{ni} = [\![w_i x_n + b_i > 0]\!]$ is the masking matrix that selects the relevant ϵ_n for each neuron i, and $\tilde{\mathbf{X}} = [\mathbf{1} \quad \mathbf{X}]$. Because the columns of $\overline{\boldsymbol{\epsilon}}$ are identical, we can swap the dot and Hadamard products:

$$
\begin{aligned}
&= \hat{\mathbf{A}}^{\top} \left(\underset{N\times 2}{\overline{\boldsymbol{\epsilon}}'} \odot \tilde{\mathbf{X}} \right) \\
&\triangleq \hat{\mathbf{A}}^{\top} \hat{\mathbf{r}},
\end{aligned}
$$

where $\overline{\boldsymbol{\epsilon}}'$ contains just 2 copies of $\hat{\boldsymbol{\epsilon}}$.

Thus, θ^*_{NN} is a critical point iff $\hat{\mathbf{r}}$ is in the null space of $\hat{\mathbf{A}}^{\top}$. We therefore proceed by analyzing $\hat{\mathbf{A}}^{\top}$. The ith row of $\hat{\mathbf{A}}^{\top}$ is given by $\mathbf{1}_i$; let us assume that the rows are sorted by the corresponding β_i, and the columns are sorted by the corresponding x_n value. Then, each row will be either consist of a block of 0s and a block of 1s. To illustrate this, consider an example with eight datapoints and five breakpoints:

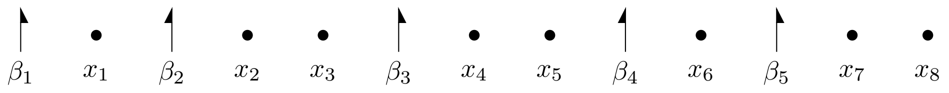

where $\bullet$ denotes a datapoint, and ⚑ denotes a breakpoint, where the direction of the "flag" indicates the facing s_i (e.g. $s_1 = +1$ so that neuron 1 is right-facing and therefore active on all data). Then, we have

$$
\hat{\mathbf{A}}_{\text{ex}}^{\top} = \begin{bmatrix}
1 & 1 & 1 & 1 & 1 & 1 & 1 & 1 \\
1 & 0 & 0 & 0 & 0 & 0 & 0 & 0 \\
0 & 0 & 0 & 1 & 1 & 1 & 1 & 1 \\
1 & 1 & 1 & 1 & 1 & 0 & 0 & 0 \\
0 & 0 & 0 & 0 & 0 & 0 & 1 & 1
\end{bmatrix}.
$$

Using the all-1s vector corresponding to the neuron active on all data, we can then apply elementary row operations to convert rows with leading 1s to have leading 0s. In our example, this gives

$$\hat{\mathbf{A}}_{\text{ex}}^{\top} = \begin{bmatrix} 1 & 1 & 1 & 1 & 1 & 1 & 1 & 1 \\ 0 & 1 & 1 & 1 & 1 & 1 & 1 & 1 \\ 0 & 0 & 0 & 1 & 1 & 1 & 1 & 1 \\ 0 & 0 & 0 & 0 & 0 & 1 & 1 & 1 \\ 0 & 0 & 0 & 0 & 0 & 0 & 1 & 1 \end{bmatrix}.$$

Because the rows are sorted by breakpoint, and each row's transition from 0s to 1s happens at the index of the first datapoint greater than that row's corresponding breakpoint, there are exactly two cases: (i) row $i + 1$ is equal to row i (when the two breakpoints land between the same pair of datapoints), or (ii) row $i + 1$ will have some additional 0s corresponding to the datapoints between the two breakpoints. If we iterate from the last row to the first and apply row-wise differences (i.e., subtracting row $i + 1$ from row i), we get for case (i) a row of all 0s, and for case (ii) a row which is 1 for each datapoint between the two breakpoints:

$$\hat{\mathbf{A}}_{\text{ex}}^{\top} = \begin{bmatrix} 1 & 0 & 0 & 0 & 0 & 0 & 0 & 0 \\ 0 & 1 & 1 & 0 & 0 & 0 & 0 & 0 \\ 0 & 0 & 0 & 1 & 1 & 0 & 0 & 0 \\ 0 & 0 & 0 & 0 & 0 & 1 & 0 & 0 \\ 0 & 0 & 0 & 0 & 0 & 0 & 1 & 1 \end{bmatrix}.$$

In this form, each row of $\hat{\mathbf{A}}^{\top}$ is given by the piece indicator $\mathbf{1}_p$, given by $\mathbf{1}_{p,n} = [\![x_n \in [\beta_p, \beta_{p+1})]\!]$. Returning to our goal of proving $\hat{\mathbf{A}}^{\top}\hat{\mathbf{r}} = \mathbf{0}$, we see that this now reduces to

$$\langle \hat{\boldsymbol{\epsilon}}_p, \mathbf{1} \rangle = 0 \quad \forall p \in [H]$$
$$\langle \hat{\boldsymbol{\epsilon}}_p, \mathbf{x} \rangle = 0 \quad \forall p \in [H],$$

which is exactly the condition that $\hat{f}(\cdot;\theta_{\text{NN}})|_{\pi_p} = m_p x + \gamma_p$ is an OLS fit of the data in piece p, for $p \in [H]$. Note that there are $P = H + 1$ pieces, but by assuming that there is at least one breakpoint active on the data (and thus outside the data), the "missing" piece is guaranteed to have no data in it, and thus vacuously be an OLS fit. Finally, the condition $\langle \hat{\boldsymbol{\epsilon}}, \mathbf{1} \rangle = 0$ can be expressed as $\sum_{p=1}^{P} \langle \hat{\boldsymbol{\epsilon}}_p, \mathbf{1} \rangle = \sum_{p=1}^{P} 0 = 0$.

Lemma 1. *For any lonely partition* Π*, there are infinitely many parameter settings* θ_{BDSO} *that induce* Π *and are global minima with* $\tilde{\ell}(\theta_{BDSO}) = 0$*. Furthermore, in the overparametrized regime* $H \geq cN$ *for some constant* $c \geq 1$*, the total number of lonely partitions, and thus a lower bound on the total number of* global *minima of* $\tilde{\ell}$ *is* $\binom{H+1}{N} = O(N^{cN})$.

PROOF. Note that each linear piece p has two degrees of freedom (slope and intercept). By way of induction, start at (say) the left-most piece. If there is a datapoint in this piece, choose an arbitrary slope and intercept that goes through it; otherwise, choose an arbitrary slope and intercept. At each subsequent piece, we can use one degree of freedom to ensure continuity with the previous piece, and use one degree of freedom to match the data (if there is any). Counting the number of lonely partitions reduces to counting the number of possible ways of allocating N datapoints (balls) into $H + 1$ pieces (urns) with at most one datapoint per piece.

B.7 The Gradient and Hessian of the Loss $\ell(\theta_{\text{NN}})$

Theorem 5. *Let θ^* be a critical point of $\ell(\cdot)$ such that $\beta_i^*(\theta^*) \neq x_n$ for all $i \in [H]$ and for all $n \in [N]$. Then the Hessian $\mathbf{H}_\ell(\theta^*)$ is the positive semi-definite Gram matrix of the set of $3H + 1$ vectors*

$$\mathcal{B} \triangleq \{v_i \mathbf{x}_i, w_i \mathbf{x}_i + b_i \mathbf{1}_i, v_i \mathbf{1}_i\}_{i=1}^H \cup \{\mathbf{1}\},$$

as shown in Equation (4). Thus, $\mathbf{H}_\ell(\theta^*)$ is positive definite iff the vectors of this set are linearly independent.

PROOF. We begin by deriving the first and second derivatives of $\hat{\mathbf{f}}$:

$$\begin{aligned}
\frac{\partial \hat{\mathbf{f}}}{\partial b_0} &= \frac{\partial}{\partial b_0}\left(\sum_{i=1}^H v_i \phi(w_i \mathbf{x} + b_i \mathbf{1}) + b_0 \mathbf{1}\right) \\
&= \mathbf{1} \\
\frac{\partial \hat{\mathbf{f}}}{\partial w_i} &= \frac{\partial}{\partial w_i}\left(\sum_{i=1}^H v_i \phi(w_i \mathbf{x} + b_i \mathbf{1}) + b_0 \mathbf{1}\right) \\
&= v_i \phi'(w_i \mathbf{x} + b_i \mathbf{1}) \odot \mathbf{x} \\
&= v_i \mathbf{x} \odot [\![w_i \mathbf{x} + b_i \mathbf{1} > 0]\!] \\
&= v_i \mathbf{x}_i
\end{aligned}$$

$$\begin{aligned}
\frac{\partial \hat{\mathbf{f}}}{\partial v_i} &= \frac{\partial}{\partial v_i}\left(\sum_{i=1}^H v_i \phi(w_i \mathbf{x} + b_i \mathbf{1}) + b_0 \mathbf{1}\right) \\
&= \phi(w_i \mathbf{x} + b_i \mathbf{1}) \\
&= (w_i \mathbf{x} + b_i \mathbf{1}) \odot [\![w_i \mathbf{x} + b_i \mathbf{1} > 0]\!] \\
&= w_i \mathbf{x}_i + b_i \mathbf{1}_i \\
\frac{\partial \hat{\mathbf{f}}}{\partial b_i} &= \frac{\partial}{\partial b_i}\left(\sum_{i=1}^H v_i \phi(w_i \mathbf{x} + b_i \mathbf{1}) + b_0 \mathbf{1}\right) \\
&= v_i \phi'(w_i \mathbf{x} + b_i \mathbf{1}) \\
&= v_i [\![w_i \mathbf{x} + b_i \mathbf{1} > 0]\!] \\
&= v_i \mathbf{1}_i
\end{aligned}$$

$$
\begin{aligned}
\frac{\partial^2 \hat{\mathbf{f}}}{\partial b_0 \partial b_0} &= \frac{\partial}{\partial b_0} \mathbf{1} = \mathbf{0} \\
\frac{\partial^2 \hat{\mathbf{f}}}{\partial w_j \partial b_0} &= \frac{\partial}{\partial w_j} \mathbf{1} = \mathbf{0} \\
\frac{\partial^2 \hat{\mathbf{f}}}{\partial v_j \partial b_0} &= \frac{\partial}{\partial v_j} \mathbf{1} = \mathbf{0} \\
\frac{\partial^2 \hat{\mathbf{f}}}{\partial b_j \partial b_0} &= \frac{\partial}{\partial b_j} \mathbf{1} = \mathbf{0} \\
\frac{\partial^2 \hat{\mathbf{f}}}{\partial b_0 \partial w_i} &= \frac{\partial}{\partial b_0} v_i \mathbf{x}_i = \mathbf{0} \\
\frac{\partial^2 \hat{\mathbf{f}}}{\partial w_j \partial w_i} &= \frac{\partial}{\partial w_j} v_i \mathbf{x}_i = \delta_{ij} v_i \mathbf{x} \odot \delta(w_i \mathbf{x} + b_i \mathbf{1}) \odot \mathbf{x} \\
&= \delta_{ij} v_i \mathbf{x}_{i,=}^{\odot 2} \\
\frac{\partial^2 \hat{\mathbf{f}}}{\partial v_j \partial w_i} &= \frac{\partial}{\partial v_j} v_i \mathbf{x}_i = \delta_{ij} \mathbf{x}_i \\
\frac{\partial^2 \hat{\mathbf{f}}}{\partial b_j \partial w_i} &= \frac{\partial}{\partial b_j} v_i \mathbf{x}_i = v_i \mathbf{x} \odot \delta(w_i \mathbf{x} + b_i \mathbf{1}) \odot \mathbf{1} \\
&= \delta_{ij} v_i \mathbf{x}_{i,=} \\
\frac{\partial^2 \hat{\mathbf{f}}}{\partial b_0 \partial v_i} &= \frac{\partial}{\partial b_0} \left(w_i \mathbf{x}_i + b_i \mathbf{1}_i \right) = \mathbf{0}
\end{aligned}
$$

$$
\begin{aligned}
\frac{\partial^2 \hat{\mathbf{f}}}{\partial w_j \partial v_i} &= \frac{\partial}{\partial w_j} \left(w_i \mathbf{x}_i + b_i \mathbf{1}_i \right) = \delta_{ij} \left[\mathbf{x}_i + (w_i \mathbf{x} + b_i \mathbf{1}) \odot \delta \left(w_i \mathbf{x} + b_i \text{[illegible]} \right. \right. \\
&= \delta_{ij} \mathbf{x}_i \\
\frac{\partial^2 \hat{\mathbf{f}}}{\partial v_j \partial v_i} &= \frac{\partial}{\partial v_j} \left(w_i \mathbf{x}_i + b_i \mathbf{1}_i \right) = \mathbf{0} \\
\frac{\partial^2 \hat{\mathbf{f}}}{\partial b_j \partial v_i} &= \frac{\partial}{\partial b_j} \left(w_i \mathbf{x}_i + b_i \mathbf{1}_i \right) = \delta_{ij} \left[\mathbf{1}_i + (w_i \mathbf{x} + b_i \mathbf{1}) \odot \delta \left(w_i \mathbf{x} + b_i \text{[illegible]} \right. \right. \\
&= \delta_{ij} \mathbf{1}_i \\
\frac{\partial^2 \hat{\mathbf{f}}}{\partial b_0 \partial b_i} &= \frac{\partial}{\partial b_0} v_i \mathbf{1}_i = \mathbf{0} \\
\frac{\partial^2 \hat{\mathbf{f}}}{\partial w_j \partial b_i} &= \frac{\partial}{\partial w_j} v_i \mathbf{1}_i = \delta_{ij} v_i \mathbf{1} \odot \delta \left(w_i \mathbf{x} + b_i \mathbf{1} \right) \odot \mathbf{1} \\
&= \delta_{ij} v_i \mathbf{1}_{i,=} \\
\frac{\partial^2 \hat{\mathbf{f}}}{\partial v_j \partial b_i} &= \frac{\partial}{\partial v_j} v_i \mathbf{1}_i = \delta_{ij} \mathbf{1}_i \\
\frac{\partial^2 \hat{\mathbf{f}}}{\partial b_j \partial b_i} &= \frac{\partial}{\partial b_j} v_i \mathbf{1}_i = \delta_{ij} v_i \mathbf{1} \odot \delta \left(w_i \mathbf{x} + b_i \mathbf{1} \right) \odot \mathbf{1} \\
&= \delta_{ij} \mathbf{1}_{i,=}
\end{aligned}
$$

where $\mathbf{1}_{i,=}$ is the vector that selects datapoints x_n that equal β_i, i.e. $\mathbf{1}_{(i,=),n} \triangleq [\![w_i x_n + b_i = 0]\!]$, and $\mathbf{x}_{i,=} \triangleq \mathbf{x} \odot \mathbf{1}_{i,=}$. Next, we calculate the first and second derivatives of ℓ. Let φ and ψ denote arbitrary individual parameters.

$$
\begin{aligned}
\ell(\mathbf{x}) &= \frac{1}{2} \langle \hat{\boldsymbol{\epsilon}}, \hat{\boldsymbol{\epsilon}} \rangle \\
\frac{\partial \ell}{\partial \varphi} &= \left\langle \hat{\boldsymbol{\epsilon}}, \frac{\partial}{\partial \varphi} \hat{\boldsymbol{\epsilon}} \right\rangle = - \left\langle \hat{\boldsymbol{\epsilon}}, \frac{\partial}{\partial \varphi} \hat{\mathbf{f}} \right\rangle \\
\frac{\partial^2 \ell}{\partial \psi \partial \varphi} &= \left\langle \frac{\partial \hat{\boldsymbol{\epsilon}}}{\partial \psi}, \frac{\partial \hat{\boldsymbol{\epsilon}}}{\partial \varphi} \right\rangle + \left\langle \hat{\boldsymbol{\epsilon}}, \frac{\partial^2 \hat{\boldsymbol{\epsilon}}}{\partial \psi \partial \varphi} \right\rangle \\
&= \left\langle \frac{\partial \hat{\mathbf{f}}}{\partial \psi}, \frac{\partial \hat{\mathbf{f}}}{\partial \varphi} \right\rangle - \left\langle \hat{\boldsymbol{\epsilon}}, \frac{\partial^2 \hat{\mathbf{f}}}{\partial \psi \partial \varphi} \right\rangle
\end{aligned}
\tag{11}
$$

Collecting and expanding all of the above together into the Hessian matrix, we have

$$\mathbf{H}_\ell \triangleq \mathrm{Hess}(\ell) \triangleq \begin{pmatrix} \ddots & & & & \\ & \frac{\partial^2 \ell}{\partial w_j \partial w_i} & \frac{\partial^2 \ell}{\partial w_j \partial v_i} & \frac{\partial^2 \ell}{\partial w_j \partial b_i} & \cdots & \frac{\partial^2 \ell}{\partial w_j \partial b_0} \\ & \frac{\partial^2 \ell}{\partial v_j \partial w_i} & \frac{\partial^2 \ell}{\partial v_j \partial v_i} & \frac{\partial^2 \ell}{\partial v_j \partial b_i} & \cdots & \frac{\partial^2 \ell}{\partial v_j \partial b_0} \\ & \frac{\partial^2 \ell}{\partial b_j \partial w_i} & \frac{\partial^2 \ell}{\partial b_j \partial v_i} & \frac{\partial^2 \ell}{\partial b_j \partial b_i} & \cdots & \frac{\partial^2 \ell}{\partial b_j \partial b_0} \\ & \vdots & & & \ddots & \vdots \\ & \frac{\partial^2 \ell}{\partial b_0 \partial w_i} & \frac{\partial^2 \ell}{\partial b_0 \partial v_i} & \frac{\partial^2 \ell}{\partial b_0 \partial b_i} & \cdots & \frac{\partial^2 \ell}{\partial b_0^2} \end{pmatrix}$$

$$= \begin{pmatrix} \ddots & & & & \\ \langle v_j \mathbf{x}_j, v_i \mathbf{x}_i\rangle - \left\langle \hat{\boldsymbol{\epsilon}}, \delta_{ij} v_i \mathbf{x}_{i,=}^{\odot 2}\right\rangle & \langle v_j \mathbf{x}_j, w_i \mathbf{x}_i + b_i \mathbf{1}_i\rangle - \langle \hat{\boldsymbol{\epsilon}}, \delta_{ij} \mathbf{x}_i\rangle & \langle v_j \mathbf{x}_j, v_i \mathbf{1}_i\rangle - \langle \hat{\boldsymbol{\epsilon}}, \delta_{ij} v_i \mathbf{1}_{i,=}\rangle & \cdots & \langle v_j \mathbf{x}_j, \mathbf{1}\rangle \\ \langle w_j \mathbf{x}_j + b_j \mathbf{1}_j, v_i \mathbf{x}_i\rangle - \langle \hat{\boldsymbol{\epsilon}}, \delta_{ij} \mathbf{x}_i\rangle & \langle w_j \mathbf{x}_j + b_j \mathbf{1}_j, w_i \mathbf{x}_i + b_i \mathbf{1}_i\rangle & \langle w_j \mathbf{x}_j + b_j \mathbf{1}_j, v_i \mathbf{1}_i\rangle - \langle \hat{\boldsymbol{\epsilon}}, \delta_{ij} \mathbf{1}_i\rangle & \cdots & \langle w_j \mathbf{x}_j + b_j \mathbf{1}_j, \mathbf{1}\rangle \\ \langle v_j \mathbf{1}_j, v_i \mathbf{x}_i\rangle - \langle \hat{\boldsymbol{\epsilon}}, \delta_{ij} v_i \mathbf{x}_{i,=}\rangle & \langle v_j \mathbf{1}_j, w_i \mathbf{x}_i + b_i \mathbf{1}_i\rangle - \langle \hat{\boldsymbol{\epsilon}}, \delta_{ij} \mathbf{1}_i\rangle & \langle v_j \mathbf{1}_j, v_i \mathbf{1}_i\rangle - \langle \hat{\boldsymbol{\epsilon}}, \delta_{ij} \mathbf{1}_{i,=}\rangle & \cdots & \langle v_j \mathbf{1}_j, \mathbf{1}\rangle \\ \vdots & & & \ddots & \vdots \\ \langle \mathbf{1}, v_i \mathbf{x}_i\rangle & \langle \mathbf{1}, w_i \mathbf{x}_i + b_i \mathbf{1}_i\rangle & \langle \mathbf{1}, v_i \mathbf{1}_i\rangle & \cdots & \langle \mathbf{1}, \mathbf{1}\rangle \end{pmatrix}$$

If we assume that $\beta_i \neq x_n$ for all i and n (which excludes a set of measure 0), we have

$$= \begin{pmatrix} \ddots & & & & \\ \langle v_j \mathbf{x}_j, v_i \mathbf{x}_i\rangle & \langle v_j \mathbf{x}_j, w_i \mathbf{x}_i + b_i \mathbf{1}_i\rangle - \langle \hat{\boldsymbol{\epsilon}}, \delta_{ij} \mathbf{x}_i\rangle & \langle v_j \mathbf{x}_j, v_i \mathbf{1}_i\rangle & \cdots & \langle v_j \mathbf{x}_j, \mathbf{1}\rangle \\ \langle w_j \mathbf{x}_j + b_j \mathbf{1}_j, v_i \mathbf{x}_i\rangle - \langle \hat{\boldsymbol{\epsilon}}, \delta_{ij} \mathbf{x}_i\rangle & \langle w_j \mathbf{x}_j + b_j \mathbf{1}_j, w_i \mathbf{x}_i + b_i \mathbf{1}_i\rangle & \langle w_j \mathbf{x}_j + b_j \mathbf{1}_j, v_i \mathbf{1}_i\rangle - \langle \hat{\boldsymbol{\epsilon}}, \delta_{ij} \mathbf{1}_i\rangle & \cdots & \langle w_j \mathbf{x}_j + b_j \mathbf{1}_j, \mathbf{1}\rangle \\ \langle v_j \mathbf{1}_j, v_i \mathbf{x}_i\rangle & \langle v_j \mathbf{1}_j, w_i \mathbf{x}_i + b_i \mathbf{1}_i\rangle - \langle \hat{\boldsymbol{\epsilon}}, \delta_{ij} \mathbf{1}_i\rangle & \langle v_j \mathbf{1}_j, v_i \mathbf{1}_i\rangle & \cdots & \langle v_j \mathbf{1}_j, \mathbf{1}\rangle \\ \vdots & & & \ddots & \vdots \\ \langle \mathbf{1}, v_i \mathbf{x}_i\rangle & \langle \mathbf{1}, w_i \mathbf{x}_i + b_i \mathbf{1}_i\rangle & \langle \mathbf{1}, v_i \mathbf{1}_i\rangle & \cdots & \langle \mathbf{1}, \mathbf{1}\rangle \end{pmatrix}$$

At a critical point of ℓ, we have $\langle \hat{\boldsymbol{\epsilon}}_i, \mathbf{x}\rangle = \langle \hat{\boldsymbol{\epsilon}}_i, \mathbf{1}\rangle = 0$, which yields further simplifications:

$$= \begin{pmatrix} \ddots & & & & \\ \langle v_j \mathbf{x}_j, v_i \mathbf{x}_i\rangle & \langle v_j \mathbf{x}_j, w_i \mathbf{x}_i + b_i \mathbf{1}_i\rangle & \langle v_j \mathbf{x}_j, v_i \mathbf{1}_i\rangle & \cdots & \langle v_j \mathbf{x}_j, \mathbf{1}\rangle \\ \langle w_j \mathbf{x}_j + b_j \mathbf{1}_j, v_i \mathbf{x}_i\rangle & \langle w_j \mathbf{x}_j + b_j \mathbf{1}_j, w_i \mathbf{x}_i + b_i \mathbf{1}_i\rangle & \langle w_j \mathbf{x}_j + b_j \mathbf{1}_j, v_i \mathbf{1}_i\rangle & \cdots & \langle w_j \mathbf{x}_j + b_j \mathbf{1}_j, \mathbf{1}\rangle \\ \langle v_j \mathbf{1}_j, v_i \mathbf{x}_i\rangle & \langle v_j \mathbf{1}_j, w_i \mathbf{x}_i + b_i \mathbf{1}_i\rangle & \langle v_j \mathbf{1}_j, v_i \mathbf{1}_i\rangle & \cdots & \langle v_j \mathbf{1}_j, \mathbf{1}\rangle \\ \vdots & & & \ddots & \vdots \\ \langle \mathbf{1}, v_i \mathbf{x}_i\rangle & \langle \mathbf{1}, w_i \mathbf{x}_i + b_i \mathbf{1}_i\rangle & \langle \mathbf{1}, v_i \mathbf{1}_i\rangle & \cdots & \langle \mathbf{1}, \mathbf{1}\rangle \end{pmatrix}$$

as desired.

Alternatively, we can start with Equation (11), and write

$$\mathbf{H}_\ell = \mathbf{G} - \langle \hat{\boldsymbol{\epsilon}}, \mathbf{H}_{\hat{f}}\rangle,$$

where $\mathbf{G}$ is the Gram matrix, and $\mathbf{H}_{\hat{f}}$ is the Hessian of $\hat{f}(x; \theta_{\mathrm{NN}})$ (with respect to θ_{NN}). Noting that $\mathbf{H}_{\hat{f}}$ is a block diagonal matrix which has only zero entries at critical points unless $\beta_i = x_n$ for some i and n yields the result.

Corollary 3. *Let θ^* be a critical point of $\ell(\cdot)$ such that its data partition is lonely, and either at least one neuron is active on all data or there is at least one pair of oppositely-faced neurons in the same data*

gap, so that $\mathbf{x} \in \mathrm{span}(\mathcal{B})$*. Then,* $\ell(\theta^*) = 0$ *and* $\mathbf{H}_\ell(\theta^*)$ *has exactly* N *non-zero eigenvalues, and thus* $3H + 1 - N$ *zero eigenvalues.*

PROOF. We begin by observing that in general, each interior interval $[x_n, x_{n+1})$ for $n \in [N-1]$ contributes at most two linearly independent feature vectors: $\mathbf{x}_i$ and $\mathbf{1}_i$ for some i. Let $\mathbf{x}_n$ and $\mathbf{1}_n$ denote the features of a right-facing neuron in the data gap (x_{n-1}, x_n). Under the assumptions that the data partition is lonely and $\mathbf{x} \in \mathrm{span}(\mathcal{B})$, we have that $\mathbf{x}_n \in \mathrm{span}(\mathcal{B})$, for all $n \in [N]$ (for any data gap that only has a left-facing neuron i, the feature $\mathbf{x}_n = \mathbf{x} - \mathbf{x}_i$). Taking pair-wise differences, we can construct the features $(0, \ldots, 0, x_n, 0, \ldots, 0)$, which form an orthogonal basis of $\mathbb{R}^N$. Thus, any further features added to $\mathcal{B}$ do not increase its span, and therefore must be linearly dependent on features already in $\mathcal{B}$, and therefore contribute only zero eigenvalues to $\mathbf{H}_\ell$.

B.8 Gradient Flow Dynamics for Spline Parameters

Theorem 6. *For a one hidden layer univariate ReLU network trained with gradient descent with respect to the neural network parameters* $\theta_{NN} = \{(w_i, b_i, v_i)\}_{i=1}^H$*, the gradient flow dynamics of the function space parameters* $\theta_{BDSO} = \{(\beta_i, \mu_i)\}_{i=1}^H$ *are governed by the following laws:*

$$\dot{\beta}_i = \frac{v_i(t)}{w_i(t)} \Big[\underbrace{\langle \hat{\boldsymbol{\epsilon}}_i(t), \mathbf{1}\rangle}_{\textit{net relevant residual}} + \beta_i(t) \underbrace{\langle \hat{\boldsymbol{\epsilon}}_i(t), \mathbf{x}\rangle}_{\textit{correlation}}\Big] \tag{12}$$

$$\dot{\mu}_i = w_i^2(t) \left[-\left(1 + \left(\frac{v_i(t)}{w_i(t)}\right)^2\right) \langle \hat{\boldsymbol{\epsilon}}_i(t), \mathbf{x}\rangle + \beta_i(t) \langle \hat{\boldsymbol{\epsilon}}_i(t), \mathbf{1}\rangle \right] \tag{13}$$

PROOF. Computing the time derivatives of the BDSO parameters and using the loss gradients of the loss with respect to the NN parameters gives us:

$$
\begin{aligned}
\frac{\partial \ell(\theta_{\mathrm{NN}})}{\partial w_i} &= v_i \langle \hat{\boldsymbol{\epsilon}}_i, \mathbf{x} \rangle \\
\frac{\partial \ell(\theta_{\mathrm{NN}})}{\partial v_i} &= \langle \hat{\boldsymbol{\epsilon}}, (w_i \mathbf{x} + b_i \mathbf{1})_+ \rangle = \langle \hat{\boldsymbol{\epsilon}}_i, w_i \mathbf{x} + b_i \mathbf{1} \rangle = w_i \langle \hat{\boldsymbol{\epsilon}}_i, \mathbf{x} \rangle + b_i \langle \hat{\boldsymbol{\epsilon}}_i, \mathbf{1} \rangle \\
\frac{\partial \ell(\theta_{\mathrm{NN}})}{\partial b_i} &= v_i \langle \hat{\boldsymbol{\epsilon}}_i, \mathbf{1} \rangle \\
\frac{\mathrm{d}\beta_i(t)}{\mathrm{d}t} &= \frac{\mathrm{d}}{\mathrm{d}t} \left(-\frac{b_i(t)}{w_i(t)} \right) \\
&= -\frac{w_i(t) \frac{\mathrm{d}b_i(t)}{\mathrm{d}t} - b_i(t) \frac{\mathrm{d}w_i(t)}{\mathrm{d}t}}{w_i(t)^2} \\
&= -\frac{w_i(t)(-\frac{\partial \ell(\theta_{\mathrm{NN}})}{\partial b_i(t)}) - b_i(t)(-\frac{\partial \ell(\theta_{\mathrm{NN}})}{\partial w_i(t)})}{w_i(t)^2} \\
&= \frac{w_i(t) \frac{\partial \ell(\theta_{\mathrm{NN}})}{\partial b_i(t)} - b_i(t) \frac{\partial \ell(\theta_{\mathrm{NN}})}{\partial w_i(t)}}{w_i(t)^2} \\
&= \frac{w_i(t) v_i(t) \langle \hat{\boldsymbol{\epsilon}}_i(t), \mathbf{1} \rangle - b_i(t) v_i(t) \langle \hat{\boldsymbol{\epsilon}}_i(t), \mathbf{x} \rangle}{w_i(t)^2} \\
&= \frac{v_i(t) \left\langle \hat{\boldsymbol{\epsilon}}_i(t), w_i(t) \mathbf{1} - b_i(t) \mathbf{x} \right\rangle}{w_i(t)^2} \\
&= \frac{v_i(t)}{w_i(t)} \left\langle \hat{\boldsymbol{\epsilon}}_i(t), \mathbf{1} - \frac{b_i(t)}{w_i(t)} \mathbf{x} \right\rangle \\
&= \frac{v_i(t)}{w_i(t)} \left\langle \underbrace{\hat{\boldsymbol{\epsilon}}_i(t),}_{\text{relevant residuals}} \quad \mathbf{1} + \beta_i(t) \mathbf{x} \right\rangle \\
&= \frac{v_i(t)}{w_i(t)} [\underbrace{\langle \hat{\boldsymbol{\epsilon}}_i(t), \mathbf{1} \rangle}_{\text{net relevant residual}} + \beta_i(t) \underbrace{\langle \hat{\boldsymbol{\epsilon}}_i(t), \mathbf{x} \rangle}_{\text{correlation}}] \\
\frac{\mathrm{d}\mu_i(t)}{\mathrm{d}t} &= \frac{\mathrm{d}}{\mathrm{d}t} w_i v_i \\
&= \frac{\mathrm{d}w_i}{\mathrm{d}t} v_i + w_i \frac{\mathrm{d}v_i}{\mathrm{d}t} \\
&= -\frac{\partial \ell(\theta_{\mathrm{NN}})}{\partial w_i} v_i - w_i \frac{\partial \ell(\theta_{\mathrm{NN}})}{\partial v_i} \\
&= -v_i^2 \langle \hat{\boldsymbol{\epsilon}}_i, \mathbf{x} \rangle - w_i^2 \langle \hat{\boldsymbol{\epsilon}}_i, \mathbf{x} \rangle - w_i b_i \langle \hat{\boldsymbol{\epsilon}}_i, \mathbf{1} \rangle \\
&= -(v_i^2 + w_i^2) \langle \hat{\boldsymbol{\epsilon}}_i, \mathbf{x} \rangle - w_i b_i \langle \hat{\boldsymbol{\epsilon}}_i, \mathbf{1} \rangle \\
&= w_i^2(t) \left[-\left(1 + \left(\frac{v_i(t)}{w_i(t)} \right)^2 \right) \langle \hat{\boldsymbol{\epsilon}}_i(t), \mathbf{x} \rangle + \beta_i(t) \langle \hat{\boldsymbol{\epsilon}}_i(t), \mathbf{1} \rangle \right]
\end{aligned}
$$

This completes the proof.

B.9 Implicit Regularization

Lemma 2. *Consider the dynamics of gradient flow on $\ell(\cdot)$ started from $\theta_{NN,\alpha}(0) \triangleq (\alpha \mathbf{w}_0, \alpha \mathbf{b}_0, \mathbf{v}_0 = \mathbf{0})$, where $w_i \neq 0 \, \forall i \in [H]$. In the limit $\alpha \to \infty$, $\boldsymbol{\beta}(t)$ does not change, i.e. each breakpoint location is fixed. In this case, the θ_{NN} model reduces to a (kernel) linear regression:*

$$\hat{\mathbf{y}} = \boldsymbol{\Phi}(\mathbf{x}; \boldsymbol{\beta})\boldsymbol{\mu} \tag{8}$$

where $\boldsymbol{\mu} \in \mathbb{R}^H$ are the regression weights and $\boldsymbol{\Phi}(\mathbf{x}; \boldsymbol{\beta}) \in \mathbb{R}^{N \times H}$ are the nonlinear features i.e. $\phi_{ni} \triangleq (x_n - \beta_i)_{s_i}$.

PROOF. First note that $\hat{f}(\cdot; \theta_{\text{NN}})$ is a 2-homogeneous model, i.e. $\hat{f}(\cdot; \lambda\theta_{\text{NN}}) = \lambda^2 \hat{f}(\cdot; \lambda\theta_{\text{NN}})$, and that $\hat{f}(\cdot; \theta_{\text{NN},\alpha}(0)) = 0$. Applying Theorem 2.2 of Chizat et al. [2019], we get that

$$\sup_{t \in [0,T]} \|\hat{f}(\mathbf{x}; \theta_{\text{NN},\alpha}(t)) - \bar{\hat{f}}(\mathbf{x}; \bar{\theta}_{\text{NN},\alpha}(t))\| = O(1/\alpha),$$

where the linearized model

$$\begin{aligned}
\bar{\hat{f}}(\mathbf{x}; \theta_{\text{NN},\alpha}) &\triangleq \hat{f}(\mathbf{x}; \theta_{\text{NN},\alpha}(0)) + \left\langle \frac{\partial \hat{f}(\mathbf{x}; \theta_{\text{NN},\alpha}(0))}{\partial \theta_{\text{NN},\alpha}}, \theta_{\text{NN},\alpha} - \theta_{\text{NN},\alpha}(0) \right\rangle \\
&= \left\langle \frac{\partial \hat{f}(\mathbf{x}; \theta_{\text{NN},\alpha}(0))}{\partial \theta_{\text{NN},\alpha}}, \theta_{\text{NN},\alpha} - \theta_{\text{NN},\alpha}(0) \right\rangle \\
&\triangleq \underset{N \times 3H}{\boldsymbol{\Phi}_{\text{NN}}} \underbrace{(\theta_{\text{NN},\alpha} - \theta_{\text{NN},\alpha}(0))}_{3H \times 1}
\end{aligned}$$

is the first order Taylor expansion of $\hat{f}(\mathbf{x}; \theta_{\text{NN},\alpha})$ about $\theta_{\text{NN},\alpha}(0)$, and $\bar{\theta}_{\text{NN},\alpha}(t)$ is the parameter trajectory of gradient flow according to the linearized model.

Next, observe that $\boldsymbol{\Phi}_{\text{NN}}$ is a matrix whose columns contain $3H$ feature vectors of the form $v_{0,i}\mathbf{x}_i$, $v_{0,i}\mathbf{1}_i$, and $w_{0,i}\mathbf{x}_i + b_{0,i}\mathbf{1}_i$ for each $i \in [H]$. Plugging these in and expanding the product, we have

$$\bar{\hat{f}}(\mathbf{x}; \theta_{\text{NN},\alpha}) = \sum_{i=1}^{H} (v_{0,i}\mathbf{x}_i)(w_i - w_{0,i}) + \sum_{i=1}^{H} (v_{0,i}\mathbf{1}_i)(b_i - b_{0,i}) + \sum_{i=1}^{H} (w_{0,i}\mathbf{x}_i + b_{0,i}\mathbf{1}_i)(v_i - v_{0,i})$$

Plugging in our $\theta_{\text{NN},\alpha}(0)$ we have $v_{0,i} = 0 \, \forall i \in [H]$, giving

$$\begin{aligned}
&= \sum_{i=1}^{H} (w_{0,i}\mathbf{x}_i + b_{0,i}\mathbf{1}_i) v_i \\
&= \sum_{i=1}^{H} w_{0,i} v_i (\mathbf{x}_i - \beta_{0,i}\mathbf{1}_i) \\
&\triangleq \boldsymbol{\Phi}(\mathbf{x}, \boldsymbol{\beta})\boldsymbol{\mu}
\end{aligned}$$

Theorem 7. *Let μ^* be the converged μ parameter after gradient flow on the BDSO model Equation* (8) *starting from $\mu_0 = \mathbf{0}$, with β held constant. Furthermore, suppose that the model perfectly interpolates the training data $\tilde{\ell}(\theta_{BDSO}) = 0$. Then,*

$$\mu^* = \arg\min_{\mu} \|\mu\|_2^2 \text{ s.t. } \mathbf{y} = \Phi(\mathbf{x}; \beta)\mu.$$

PROOF. This proof follows the same proof strategy as the proof of Theorem 1 of Woodworth et al. [2020]. First, we consider the dynamics of $\mu(t)$ under gradient flow:

$$\dot{\mu}(t) = -\frac{\partial \tilde{\ell}}{\partial \mu} = -\left\langle \hat{\epsilon}(t), \frac{\partial \hat{\epsilon}(t)}{\partial \mu} \right\rangle = \langle \hat{\epsilon}(t), \Phi \rangle .$$

Note that only the first term varies with time. Using this, we can integrate over time to get

$$\mu^* = \mu(\infty) = \mu_0 + \int_0^\infty \langle \hat{\epsilon}(t), \Phi \rangle \, \mathrm{d}t = \left\langle \int_0^\infty \hat{\epsilon}(t) \, \mathrm{d}t \,, \Phi \right\rangle \triangleq \langle \mathbf{r}^*, \Phi \rangle \tag{14}$$

where we define $\mathbf{r}^* \triangleq \int_0^\infty \hat{\epsilon}(t) \, \mathrm{d}t$.

Next, consider the convex optimization problem

$$\arg\min_{\mu} Q(\mu) \text{ s.t. } \Phi\mu = \mathbf{y},$$

where $Q(\cdot)$ is some as-yet unspecified convex real-valued function. Then, the KKT conditions for this problem are

$$\begin{gathered} \Phi\mu = \mathbf{y} \\ \exists \nu^* \text{ s.t. } \nabla_\mu Q(\mu^*) = \left\langle \nu^*, \nabla_\mu \hat{\mathbf{f}}(\mu^*) \right\rangle = \langle \nu^*, \Phi \rangle . \end{gathered} \tag{15}$$

If we set $\nu^* = \mathbf{r}^*$, then the right hand sides of Equations (14) and (15) are equal, implying that the left hand sides are also equal:

$$\nabla_\mu Q(\mu^*) = \mu^*.$$

Integrating both sides with respect to each μ_i, we have

$$Q(\mu) = \sum_{i=1}^H \int_0^{\mu_i} \mu_i \, \mathrm{d}\mu_i = \sum_{i=1}^H \frac{1}{2}\mu_i^2 \propto \|\mu\|_2^2$$

$$\hat{f}''(x; \theta_{\text{BDSO}}) = \mu(x,+)p_H(x,+) - \mu(x,-)p_H(x,-)$$

Lemma 4. *Let $(\theta_{BDSO,H})_H \triangleq ((\mu_{i,H}, \beta_{i,H}, s_{i,H})_{i=1}^H)_H$ be a sequence of width-H parameter settings such that the breakpoints $(\beta_{i,H})_{i=1}^H$ are evenly spaced on the interval $[a,b]$ for every H, $\lim_{H\to\infty} \mu_{i,H} = 0$ for every i, and $\theta_{BDSO,\infty} \triangleq \lim_{H\to\infty} \theta_{BDSO,H}$ is well-defined. Let $\hat{f}_\infty(\cdot; \theta_{BDSO,\infty}) \triangleq \lim_{H\to\infty} \hat{f}(\cdot; \theta_{BDSO,H})$.*

Then,

$$\hat{f}''_\infty(x;\theta_{BDSO,\infty}) = \lim_{H\to\infty} \frac{\mu_{i(x),H}}{\Delta\beta_{i(x),H}},$$

where $(\mu_{i(x),H})_H$ *is the sequence of delta-slopes corresponding to the unique sequence* $(\beta_{i(x),H})_H$ *of breakpoints that converge to* x*, and* $\Delta\beta_{i(x),H} \triangleq \beta_{p(i(x))+1,H} - \beta_{p(i(x)),H}$.

PROOF. From Equation (1), we can derive

$$\hat{f}'(x;\theta_{\text{BDSO},H}) = \sum_{i=1}^{H} \mu_i \begin{cases} [\![x > \beta_i]\!], & s_i = 1 \\ [\![x < \beta_i]\!], & s_i = -1 \end{cases} \tag{16}$$

$$\hat{f}''(x;\theta_{\text{BDSO},H}) = \sum_{i=1}^{H} \mu_i s_i \delta(x - \beta_i). \tag{17}$$

For convenience, let β_p be the pth smallest β_i:

$$= \sum_{p=1}^{H} \mu_p s_p \delta(x - \beta_p)$$

Multiplying each term by $\frac{\Delta\beta_p}{\Delta\beta_p} = 1$, we get

$$= \sum_{p=1}^{H} \Delta\beta_p \frac{s_p \mu_p}{\Delta\beta_p} \delta(x - \beta_p)$$

Next, note from Equations (16) and (17) that the term $s_p\mu_p$ is exactly the change in $\hat{f}'(\cdot;\theta_{\text{BDSO},H})$ at β_p. Plugging this in, we get:

$$= \sum_{p=1}^{H} \Delta\beta_p \left[\frac{\hat{f}'(x + \frac{\Delta\beta_p}{2}) - \hat{f}'(x - \frac{\Delta\beta_p}{2})}{\Delta\beta_p} \right] \delta(x - \beta_p)$$

Taking the $H \to \infty$ limit, we note that the overall expression has the form of a Riemann sum, and the bracketed expression is the definition of $\hat{f}''_\infty(\cdot;\theta_{\text{BDSO},\infty})$, and is well-defined because $(\mu_{i,H})_H \to 0$ by hypothesis:

$$\xrightarrow{H\to\infty} \int_a^b \hat{f}''_\infty(\beta, \theta_{\text{BDSO},\infty}) \delta(x - \beta)\, \mathrm{d}\beta$$
$$= \hat{f}''_\infty(x, \theta_{\text{BDSO},\infty})$$

Corollary 4. *Consider the setting of Theorem 7, with the additional assumption that the breakpoints are uniformly spaced, and let* $H \to \infty$*. Then the learned function* $\hat{f}_\infty(x; \boldsymbol{\mu}^*, \boldsymbol{\beta})$ *is the global minimizer of*

$$\inf_f \int_{-\infty}^{\infty} f''(x)^2\, \mathrm{d}x \;\text{ s.t. } y_n = f(x_n)\ \forall n \in [N],$$

As such, $\hat{f}(x; \boldsymbol{\mu}^*, \boldsymbol{\beta}^*)$ *is a natural cubic smoothing spline with* N *degrees of freedom Ahlberg et al. [1967].*

PROOF. Take the limit as $H \to \infty$ of each step in the proof of Theorem 7 that requires it. This amounts to making the replacements

$$\begin{aligned} \boldsymbol{\mu} \in \mathcal{H}_H \equiv \mathbb{R}^H &\mapsto \mu(\cdot) \in \mathcal{H} \equiv \mathcal{L}_2([a,b]) \\ \langle \cdot, \cdot \rangle_{\mathcal{H}_H} &\mapsto \langle \cdot, \cdot \rangle_{\mathcal{H}} \\ \frac{\partial}{\partial \boldsymbol{\mu}} &\mapsto \frac{\delta}{\delta \boldsymbol{\mu}(\cdot)} \\ \min &\mapsto \inf \\ \|\boldsymbol{\mu}\|_2^2 &\mapsto \int_{-\infty}^{\infty} \hat{f}''(x)^2 \, \mathrm{d}x \end{aligned}$$

For the last replacement, use Lemma 4 to see that minimizing

$$\begin{aligned} \|\boldsymbol{\mu}\|_2^2 &= \sum_{i=1}^{H} \mu_i^2 \\ &\propto \frac{1}{\Delta\beta} \sum_{i=1}^{H} \mu_i^2 \\ &= \frac{1}{\Delta\beta} \sum_{i=1}^{H} (s_i \mu_i)^2 \\ &= \sum_{i=1}^{H} \frac{(s_i \mu_i)^2}{\Delta\beta_i} \\ &\propto \sum_{i=1}^{H} \left(\frac{s_i \mu_i}{\Delta\beta_i} \right)^2 \end{aligned}$$

is equivalent to minimizing $\int_{-\infty}^{\infty} \hat{f}''(x)^2 \, \mathrm{d}x$ in the infinite width limit.
